# Chromosome level assembly and secondary metabolite potential of the parasitic fungus *Cordyceps militaris*

**DOI:** 10.1186/s12864-017-4307-0

**Published:** 2017-11-25

**Authors:** Glenna J. Kramer, Justin R. Nodwell

**Affiliations:** Department of Biochemistry, University of Toronto, MaRS Centre, West Tower, 661 University Avenue, Toronto, ON M5G 1M1 Canada

**Keywords:** *Cordyceps militaris*, Entomopathogenic fungi, Genome, SMRT sequencing, Secondary metabolite

## Abstract

**Background:**

*Cordyceps militaris* is an insect pathogenic fungus that is prized for its use in traditional medicine. This and other entomopathogenic fungi are understudied sources for the discovery of new bioactive molecules. In this study, PacBio SMRT long read sequencing technology was used to sequence the genome of *C. militaris* with a focus on the genetic potential for secondary metabolite production in the genome assembly of this fungus.

**Results:**

This is first chromosome level assembly of a species in the *Cordyceps* genera. In this seven chromosome assembly of 33.6 Mba there were 9371 genes identified. *Cordyceps militaris* was determined to have the MAT 1-1-1 and MAT 1-1-2 mating type genes. Secondary metabolite analysis revealed the potential for at least 36 distinct metabolites from a variety of classes. Three of these gene clusters had homology with clusters producing desmethylbassianin, equisetin and emericellamide that had been studied in other fungi.

**Conclusion:**

Our assembly and analysis has revealed that *C. militaris* has a wealth of gene clusters for secondary metabolite production distributed among seven chromosomes. The identification of these gene clusters will facilitate the future study and identification of the secondary metabolites produced by this entomopathogenic fungus.

**Electronic supplementary material:**

The online version of this article (doi:10.1186/s12864-017-4307-0) contains supplementary material, which is available to authorized users.

## Background

**Fig. 1:**
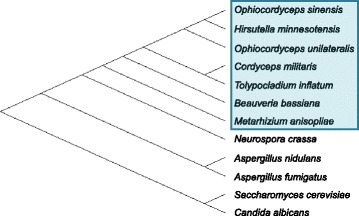
Phylogenetic tree showing evolutionary relationships between common fungal species and insect pathogenic species, including *Cordyceps militaris*, the species of interest in this study. Insect pathogenic fungi are highlighted by a blue box

Entomopathogenic fungi are a fascinating group of insect parasitic microbes, which include species from a variety of different fungal taxa including *Beauvaria*, *Hirsutella*, *Metarhizium Cordyceps and Ophiocordyceps* (Fig. 1). These entomopathogenic fungi typically have a lifecycle in which the host is infected and is killed in the process of fungal propagation. Two closely related genera of entomopathogemic fungi, *Cordyceps* and *Ophiocordyceps* (often times just referred to as cordyceps in common literature) are characterized by their unique lifecycle and a specific process by which they parasitize and reproduce using the insect host.

Fungal spores and hyphae are able to penetrate insect cuticle and then colonize and proliferate within their body cavity. As the insect’s body is used as a nutrient reservoir for growth, the insect’s behaviour is modified, eventually leading the host to die in an advantageous location for fungal spore dispersal. The fungus then emerges as a fruiting body from the corpse of the insect, which matures and disperses spores of the next generation. Spread globally, *Cordyceps* and *Ophiocordyceps* fungi have been described in climates across Asia, the Americas, Europe and Australia, with many of these species having not been characterized. Although these genera are believed to contain well over 400 species of fungi, there are a few standout examples which are revered for their medicinal potential or unusual host pathogenesis.

*Ophiocordyceps sinensis*, found in the mountains of Tibet, infects and kills ghost moth larvae to give the highly prized herbal remedy “dong chong xia cao,” which is believed to treat a plethora of disorders [1]. This prized specimen is identified by the fruiting body growing from the ground, as the infected ghost moth larvae dies situated just below the surface of the soil with its head oriented upward, from which the fruiting body emerges. *Ophiocordyceps unilateralis*, also known as the zombie-ant fungus, is noted for its pathogenic process in ants, which is characterized by particular behaviour modifications in the host, that leaves the host ant perished with its jaw clamped to a leaf in prime location for spore dispersal [2]. *Cordyceps militaris*, which is a common component of supplements as it is also believed to have medicinal potential, is often used as a cheaper and more readily available version of *Ophiocordyceps sinensis* [3, 4].

The genome of a handful of these fungi have been sequenced, however, the available assemblies are often fragmented in over 500 contigs [5–8]. These assemblies do indicate that these fungi are capable to producing natural products, possibly over 30 distinct molecules per species. Only a few of these natural products from entomopathogenic fungi have been isolated and described, including the immunosuppressant, cyclosporine, from *Tolypocladium inflatum*, and fingolimoid, the immunomodulatory molecule derived from the *Isaria sinclarii* natural product myriosin, signifying that these fungi may be an underexplored source of novel molecules [9, 10].

Natural products have been established as a source of bioactive molecules, however, discovery has dwindled implying the need for new sources. Fungi have been shown to produce a wealth of diverse molecules [11] suggesting that *Cordyceps* and *Ophiocordyceps* could be an understudied and relevant avenue for the discovery of natural products. Furthermore, by studying the secondary metabolites in *Cordyceps and Ophiocordyceps* not only is there the potential for discovery of new bioactives, but for the identification and study of chemicals that have a role in the process of host pathogenesis, from behaviour modifying molecules to insecticides [12]. As genome sequencing becomes cheaper, faster, and more readily available, the possibility of taking a computational approach to genome mining for secondary metabolite discovery in fungi becomes a more realistic possibility, allowing for the study of secondary metabolites which may be cryptic under typical laboratory conditions [13]. Indeed, laboratory culture of these organisms is a challenge due to their slow growth rates – a genome-based approach to their natural product genes is likely essential for this field to progress.

In this study, a new method of long read sequencing, Pacific Biosciences SMRT sequencing is applied to an exemplary sample from the *Cordyceps* genera, *Cordyceps militaris*, a strain isolated from butterfly pupa. The overarching goal is to provide a chromosome level genome assembly to serve as a model for the genera. Furthermore, as these fungi have the potential to produce many understudied natural products, this study is focused on the genetic potential for secondary metabolite expression in this organism.

## Results

### General genome features

Purified genomic DNA isolated from culture of *C. militaris* grown up from a single colony was sequenced using the Pacific Biosciences platform using a sheared large insert library [14]. Sequence data from 6 SMRT cells, providing approximately 180× coverage were assembled using two de novo assemblers, Celera with the PBcR protocol and the HGAP2 protocol from SMRT portal [15–17]. Both chosen assemblers were applied to self-correct the reads, a process in which the shorter PacBio reads were used to error correct the long PacBio reads. These corrected reads were then subsequently assembled into contigs. The PBcR-pipeline gave an assembly with 32 contigs, four of which had telomeric repeats (CCCTAA or TTAGGG)_n_ on either the 5′ or 3′ end of the contig.

The second assembly protocol, the HGAP2 protocol from the SMRT portal software package, which also included an additional polishing step using the Quiver algorithm, yielded an even further improved assembly. This assembly, which contained 18 contigs, gave five assembled chromosomes, having telomeric repeats (CCCTAA or TTAGGG)_n_ on both the 5′ and 3′ ends of the sequence and four having telomeric repeats on one of the 5′ or 3′ end of the sequence. After manually curating the assembly and submitting the curated assembly to the SMRT resequencing protocol, the resultant assembly contained 7 contigs, each with telomeres on both ends, indicative of 7 chromosomes. Coverage across these seven chromosomes, including regions where the assembly was manually curated is shown in Additional file 1: Figure S1. The coverage across the chromosomes is generally consistent in the assembly after manual curation and the SMRT resequencing protocol. The N_50_ (5.78kba) and N_max_ (8.29kba) remain unchanged when comparing the initial assembly and the curated and resequenced assembly. However, there is a spike in coverage in the contig corresponding to chromosome IV, possibly implying a collapsed repeat region within the assembly (Additional file 1: Figure S1D). When this area of the assembly is further inspected, it is noted that there are a large number of short low quality repeated reads, which align on top of >30× coverage of high quality reads that span this repetitive region. The repetitive sequence of this region and the overabundance of low quality reads in this region was noted prior to manual curation. The initial assembly consisted of 3 contigs containing these short repetitive reads, each about 30,000 bp in length, which overlapped in this region. With an overall coverage of approximately 150×, a genome size of 33.6 Mba, and a GC content of 50.9%. (Table 1), the BUSCO completeness of this assembly is 98.2% with only 0.4% missing [18]. Furthermore, the assembly is comparable to the previously sequenced *C. militaris* Cm01 (though that sequence is broken into a very large number of contigs) with a similar genome size of 32.2 Mba and a GC content of 51.4% [6].

**Table 1:** Main features of *C. militaris* genome assembly

Main Features of *C. militaris* Genome Assembly	
Genome size (Mba)	33.6
Number of chromosomes	7
Fold coverage	149.5×
GC content	50.9

The seven assembled *C. militaris* chromosomes range in size from 1.9 to 8.3 Mba. The sequenced haploid genomes of *Aspergillus niger* and *Neurospora crassa* contain eight and seven chromosomes, respectively [19, 20]. Furthermore, a karyotype analysis of *Tolypocladium inflatum* shows that this related species contains seven chromosomes ranging in size from 1.0 to 6.3 Mba [21], suggesting that the assembly with seven chromosomes is reasonable for *C. militaris*.

The MAKER genome annotation pipeline [22, 23] predicted 9907 genes for *C. militaris*. Passing the MAKER gene predictions through an additional evidence modeler using Funannotate gave a set of 9371 genes with a BUSCO analysis of the resulting gene set estimating a completeness of 93.7% with 3.9% of genes missing [18, 24]. Estimates of mean gene length, mean exon length, mean intron length and gene density (Table 2) are similar to those of *C. militaris* Cm01 and other filamentous ascomycete fungi (Table 3) [5–7, 12, 25, 26]. An Interpro analysis of the annotated genes using the Blast2GO suite was used to assign 8792 genes (93.8%) of genes InterPro IDs. A Gene Ontology (GO) annotation was assigned to 6453 of the genes (68.9%).

**Table 2:** Features of *C. militaris* genome annotation

Main Features of *C. militaris* Genome Annotation	
Genome Size (Mba)	33.6
Number of Chromosomes	7
Number of genes	9371
Number of exons	26,128
Number of introns	16,759
Total gene length (Mba)	16.1
Mean gene length (bases)	1724
Gene density (genes per Mba)	278.9
Mean exon length (bases)	548
Mean intron length (bases)	111
Mean introns per gene (bases)	1.8
Genome coding (%)	48.0

**Table 3:** General features of ascomycetes related to *C. militaris*

	*Cordyceps militaris* ATCC® 34164	*Cordyceps militaris* Cm01	*Tolypocladium inflatum*	*Fusarium graminearum*	*Ophiocordyceps sinensis*	*Ophiocordyceps unilateralis*
Genome Size (MBa)	33.6	32.2	30.35	36.09	116.42	26.1
GC Content (%)	50.9	51.4	58.0	48.3	43.1	54.8
Predicted Genes	9371	9684	9998	13,321	7939	7831
Gene density (Genes/Mbp)	278.9	301	329	369	69	301
Mean gene length	1724	1742	1670	1583	1693	1420^a^
Mean exon length	548	507	570	508	NR	261^a^
Mean intron length	111	98	78	68	103	62^a^
Mean introns per gene	1.8	2.0	2.2	2.2	NR	3^a^

^a^Values presented as a median as opposed to a mean, NR is not reported in original publication

### Mating type loci

**Fig. 2:**
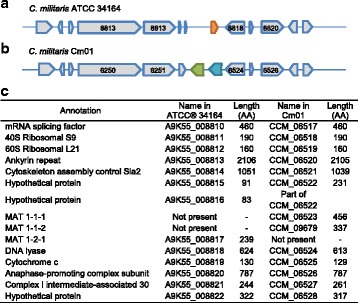
Comparison of mating type loci and surrounding genes in *C. militaris* ATCC® 34164 and *C militaris* Cm01. MAT 1-1-1 gene is shown in green, MAT 1-1-2 gene is shown in cyan, MAT 1-2-1 gene is shown in orange. Select genes are numbered for reference in the figure. Gene names from left to right are also listed in corresponding table. **a** *C. militaris ATCC® 34164*. **b** *C. militaris* Cm01. **c** Table of potential gene function and name

The sequence of our isolate revealed the presence of only a MAT 1-2-1 mating type gene present on chromosome VII, supporting the notion that this fungus is indeed heterothalic. Both MAT 1-1, MAT 1-2 and strains with hybrid mating loci have been reported [6]. The previously sequenced *C. militaris* Cm01 strain was determined to have both MAT 1-1-1 and MAT 1-1-2 mating type genes. The *C. militaris* Cm06 strain was reported to be a hybrid strain containing both the MAT 1-1 and MAT 1-2 mating types, with single spore isolates from this hybrid strain producing progeny with either the MAT 1-1 (93.3%) or MAT 1-2 (6.7%) loci [6]. Both the MAT 1-1 and MAT 1-2 containing isolates have been shown to fruit, but only the hybrid strain containing both the MAT 1-1 and MAT 1-2 loci was able to produce mature spores [6]. However, fruiting bodies were not observed under analogous conditions with the ATCC® 34164 strain. Comparison of the genomic regions containing the mating type genes in both our ATCC® 34164 strain and the Cm01 strain reveal that the genes in these regions are highly similar, with the exception of the MAT genes (Fig. 2).

### Cordycepin

One hallmark molecule of interest in *C. militaris* is the nucleoside analogue cordycepin. Although this biosynthesis is unknown, it is proposed that this mechanism is dependent upon a reduction step, believed to be potentially catalyzed by a ribonucleoside diphosphate reductase (RNR) [6, 27, 28]. However, our sequenced *C. militaris* is similar to the sequenced Cm01 strain, in that it only seems to possess two type I RNRs (genes A9K55_000536 and A9K55_003140), both of which have homologues in non-cordycepin producing fungus, and have been identified in *C. militaris* Cm01, leaving the biosynthesis of cordycepin elusive [6].

### Secondary metabolite potential

Sequenced fungi from the *Cordyceps*, *Ophiocordypces*, and related genera have revealed the potential for production of over 30 diverse secondary metabolites per strain [7, 8, 26, 29, 30]. The limited number of prior systematic studies to identify bioactive secondary metabolites from *Cordyceps* and related fungi have shown that a number of novel molecules can be produced by these microbes [31–35]. However, these studies do not nearly capture the full secondary metabolite potential of these fungi, likely due to the fact that many of these metabolites may be cryptic and not expressed under the tested laboratory conditions. To determine whether this fungus could produce a wealth of secondary metabolites, the genetic potential for diverse metabolite production became the focus of the study. Using two gene cluster predictors, AntiSMASH and SMURF, all seven *C. militaris* chromosomes were profiled for the presence of genes that could be responsible for the biosynthesis of secondary metabolites [36–38]. The fungal version of AntiSMASH, predicted 32 secondary metabolites and SMURF predicted 25. Taken together, the two algorithms predicted the presence of 36 unique gene clusters which could be responsible for secondary metabolite production in *C. militaris* (Table 4).

**Table 4:** Number of natural product clusters predicted by the AntiSMASH and SMURF gene finding algorithms per chromosome and the number of unique natural products (NP) predicted in total by comparing the results of both algorithms

Chromosome	AntiSMASH	SMURF	Unique NP
I	1	1	1
II	3	1	4
III	4	4	5
IV	3	1	4
V	6	6	7
VI	8	6	8
VII	7	6	7
Total	32	25	36

**Fig. 3:**
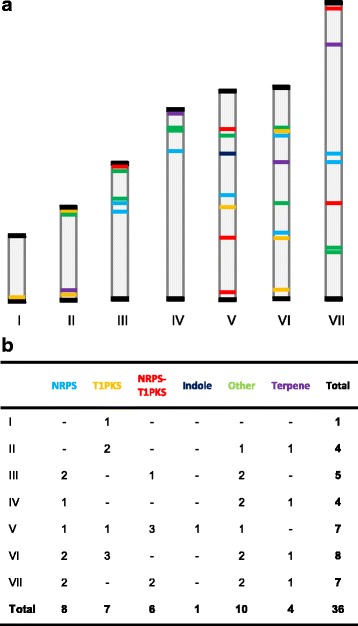
Putative natural products in *C. militaris*. The classes of natural products are denoted by the following colors NRPS (cyan), T1PKS (orange), T1PKS-NRPS (red), Indole (indigo), Other (green) Terpene (violet). **a** The distribution of natural product producing gene clusters on the chromosomes of *C. militaris*. **b** The number of natural product gene cluster on each chromosome, grouped by class

Distribution of these secondary metabolite producing genes were mapped on the 7 chromosomes (Fig. 3a). No gene clusters for secondary metabolites were noted in the presumably collapsed area of the genome shown in chromosome IV. The 36 metabolite producing gene clusters were from a variety of classes, including eight nonribosomal peptide synthetases (NRPS), seven type 1 polyketide synthases (T1PKS), six polyketide synthase-nonribosomal peptide synthetase (PKS-NRPS) hybrids, four terpenes, one indole and ten falling into other classes (Fig. 3b).

For comparison with the previously sequenced strain, the antiSMASH algorithm was also used to predict the presence of natural product producing gene clusters in the in *C. militaris* Cm01. In Cm01 there were 28 natural product clusters identified by AntiSMASH, compared to the 32 in our strain of study. All of the 28 clusters were present in the ATCC® 34164 strain and the ATCC®34164 strain had four additional natural product clusters identified. These additional clusters are predicted to produce one indole (V-3), two T1PKS (VI-2, VI-8) and one T1PKS-NRPS (VII-5). Furthermore, using the ClusterFinder algorithm in the fungal version AntiSMASH, an additional 41 putative clusters were predicted, bringing the total to 73 predicted clusters from AntiSMASH with ClusterFinder, suggesting that the secondary metabolite potential of this organism is impressive.

## Discussion

Herein is described the first chromosome level assembly of a *Cordyceps* genome. This seven chromosome assembly has revealed that this heterothallic strain, which contains 9371 genes, is capable of producing a wealth of secondary metabolites. Of the 36 gene clusters identified in ATCC®34164 by both the antiSMASH and SMURF algorithms (Additional file 2: Table S1), 3 clusters, III-1, V-6 and VII-5, are of particular interest as they have homology with gene clusters from other organisms that produce characterized natural products [39, 40].

**Fig. 4:**
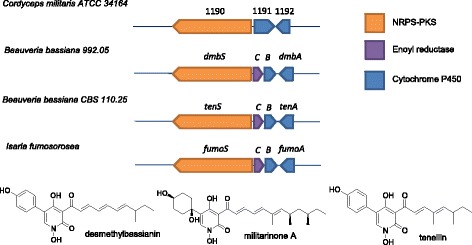
Comparison of gene clusters producing a 2-pyridone alkaloid molecule to the genes A9K55_001190 to A9K55_001192 in cluster III-1 in *C. militaris* ATCC ® 34164. The desmethylbassianin cluster (*dmb*), tenellin (*ten*), and fumosorone (*fum*) clusters are shown. Genes are color coded by function. Structures of 2-pyridone alkaloids are displayed

It seems that a logical product of cluster III-1 could be a 2-pyridone alkaloid molecule. The hybrid NRPS-PKS central to this cluster (A9K55_001190) is similar to the NRPS-PKS responsible for the production of desmethylbassianin (70% identity) and tenellin (67% identity) from *Beauveria bassiana* and fumosorinone (66% identity) from *Isaria fumosoinone* [41–43]. Both the desmethylbassanin and tennellin gene clusters consist of 4 genes: the NRPS-PKS hybrid, an enoyl reductase and two cytochrome P450s (Fig. 4). In *C. militaris*, based on the sequence of gene A9K55_001191 it seems that the missing enoyl reductase may be fused with the cytochrome P450. Interestingly, a structurally related pigmented derivative, militarinone A, and the variants militarinone B–D, have been isolated from the possible *C. militaris* anamorph, *Paecilomyces militaris* [44, 45]. However, militarinone A–D was not identified by mass in extracts of the *C. militaris* strain of interest in this study.

**Fig. 5:**
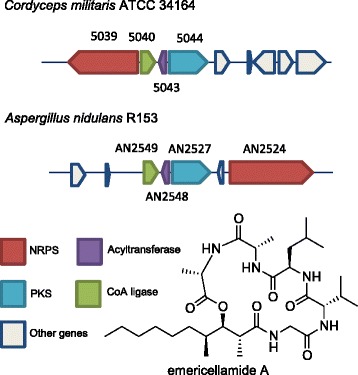
Comparison of genes A9K55_005039, A9K55_005040, A9K55_005043 and A9K55_005044 in *C. militaris* ATCC® 34164 to an emericellamide producing gene cluster in *Aspergillus nidulans*. Genes are color coded by function. The structure of emericellamide is displayed

On chromosome V, a cluster (V-6) with homology to the emercellamide producing cluster is present (Fig. 5). The emercellamide family molecules produced from the hybrid NRPS-PKS containing cluster have been described in the marine fungus *Emericella*, as well as the fungus *Aspergillus nidulans* [46, 47]. Other related molecules, scopularide A and W493-B have been isolated from *Scopulariopsis brevicaulis* and *Fusarium pseudograminearum*, respectively [48–50]. The biosynthesis of emericellamide A in *Aspergillus* has been described and is shown to rely on four genes [47]. Comparing this emercellamide-like cluster in *C. militaris* to the gene cluster producing emercellamide in *Aspergillus* shows a conservation of 4 genes: an NRPS, a PKS, an acyl-transferase and a CoA ligase. The NRPS present in *C. militaris* (A9K55_005039) has 98% coverage and 43% identity with the NRPS in *Aspergillus nidulans*.

**Fig. 6:**
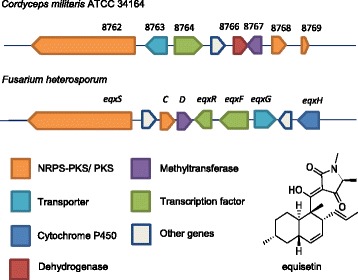
Comparison of genes A9K55_008762 to A9K55_008769 in *C. militaris* ATCC® 34164 to an equisetin producing cluster in *Fusarium heterosporum*. Genes are color coded by function. The structure of equisetin is displayed

A cluster with similarities to the equisitin-producing cluster (VII-5) is also present in *C. militaris* on chromosome VII. This molecule, equisetin, was described as having structural similarities to the cholesterol lowering molecule lovastatin and was first isolated from *Fusarium equiseti* with a described bioactivity as a HIV-1 integrase inhibitor [51–53]. The biosynthesis, studied in *Fusarium heterosporum*, reveals that the gene cluster consists of seven genes, two NRPS/PKS, two regulators, an oxidase, a methyltransferase and a transporter [53]. The comparable cluster in *C. militaris* consists of five genes, homologous to the genes present in the *F. heterosporum* minus one of the regulators and the oxidase (Fig. 6). The NRPS/PKS present in *C. militaris* (A9K55_008762) has 99% coverage and 50% identity with the NRPS/PKS in *Fusarium heterosporum* and seems to be well conserved among fungi in the *Aspergillus* and *Penicillum* genera.

This assembly of a genome from the *Cordyceps* genera, *Cordyceps militaris*, has shown the potential for production of an array of potentially novel natural products. This species is predicted to produce at least 36 secondary metabolites, three of which have significant similarity to characterized gene clusters. As fungal secondary metabolites can be cryptic under standard laboratory conditions, this assembled genome will allow for the application of genome mining techniques to guide the discovery and identification of new natural products. This can progress forward through a variety of techniques; one approach is to heterologously express gene clusters identified in the *C. militaris* genome. Alternatively, utilizing the genome to extrapolate potential natural products for expression can give important clues about the structure and favorable culture conditions of a secondary metabolite associated with a characterized gene cluster. This knowledge can increase the likelihood of the production of the correlated molecule and simplify structural determination. Regardless, this assembly has shown that there is a great potential for the production of secondary metabolites in *C. militaris* and that this and other fungi from related *Cordyceps* and *Ophiocordyceps* genera could provide a wealth of molecular structural diversity.

## Conclusions

Presented here is the first chromosome level assembly of a genome from the *Cordyceps* genera. This assembly and analysis has revealed that *C. militaris* has seven chromosomes containing a wealth of gene clusters for secondary metabolite production. Of the 36 gene clusters identified using the antiSMASH and SMURF algorithms, three clusters are found to have a high degree of similarity with clusters from other organisms that produce a known molecule. With this genome, further study and characterization of the secondary metabolites produced by *C. militaris* can be aided through genome based techniques including heterologous expression of gene clusters. As there is great potential for the production of secondary metabolites from *C. militaris*, this is one step towards discovering and characterizing the wealth of molecular structural diversity in this genera.

## Methods

### Phylogenetic tree construction

To compare the fungal species of interest, 18S rRNA sequences were obtained from the Silva database [54]. Sequence alignment was performed using ClustalW [55]. To construct the phylogenetic tree, The evolutionary history was inferred using the Neighbor-Joining method [56]. The optimal tree with the sum of branch length = 0.35499733 is shown. The evolutionary distances were computed using the Maximum Composite Likelihood method [57] and are in the units of the number of base substitutions per site. The analysis involved 12 nucleotide sequences. Codon positions included were 1st + 2nd + 3rd + Noncoding. All ambiguous positions were removed for each sequence pair. There were a total of 988 positions in the final dataset. Evolutionary analyses were conducted in MEGA7 [58].

### Fungal strain and maintenance

*Cordyceps militaris* ATCC® 34164 was received from the American Type Culture Collection (ATCC). This strain, as described in the ATCC records was isolated from a butterfly pupa. Fungal cultures were maintained at 23.0 °C on potato dextrose agar. The nrDNA of extracted genomic DNA was amplified using the ITS4 and ITS5 primer pairs, sequenced and compared against the BLAST database to determine sample validity [59–61].

### Genomic DNA extraction and purification

Liquid cultures, containing 5 mL of seed media (10 g peptone, 40 g maltose, 10 g yeast extract, 1 g agar in 1 L DI water) in a culture tube were aseptically inoculated with a 3 mm square agar slab containing mycelial growth. The fungal mycelia were grown for 5 days at 23.0 °C. The mycelial mat was harvested, rinsed with sterile TE buffer and then frozen in liquid nitrogen. Frozen mycelia were macerated with a mortar and pestle with a spatula tip of aluminum oxide to aid in grinding the sample. The mycelial powder was transferred to a set of epitubes and 500 μL of CTAB DNA extraction buffer was added (100 mM Tris pH = 8.0, 10 mM EDTA, 2% CTAB, 2.8 M NaCl). The samples were incubated at room temperature for 5 min, then 2 μL of RNAse A (Thermo Scientific, 10 mg/mL) and 10 μL of Proteinase K were added (Invitrogen, 10 mg/mL) and inverted to mix. After centrifuging for 5 min, the pellet was ground in the epitube with a pellet pestle, then incubated for an additional 5 min before purification with phenol-chloroform. Each sample was washed twice with phenol-chloroform (50:50, phenol buffered with Tris pH 8.0, 600 μL) then twice with chloroform (600 μL). The resulting DNA containing aqueous portions were pooled and DNA was precipitated using cold ethanol (2.5× sample volume) and 3 M sodium acetate (0.1× sample volume). DNA was precipitated for at least 30 min by storing at −20 °C. The DNA precipitate was collected by centrifuging for 30 min, the pellet was washed with 70% ethanol and resuspended in TE buffer. The DNA was further purified with AMPure XP beads (Agencourt) by using an equal volume of beads to volume of DNA and eluting into TE. DNA was quantified using a PicoGreen assay (ThermoFisher) prior to sequencing.

### Genome sequencing

The *Cordyceps militaris* DNA was sequenced using Pacific Biosciences RS II sequencing at the Genome Quebec Innovation Center (McGill University, Montreal, Canada). The sample was prepared using a large insert sheared DNA library and was sufficient for sequencing 6 SMRT cells.

### Genome assembly

The sequencing reads were assembled using two different assemblers. The first assembler chosen was the PBcR pipeline from the Celera assembler (version 8.3rc2) using a genome size of 32 Mba [16]. The second assembler was SMRT portal (version 2.3.0) launched from an Amazon machine image. Assembly was performed on all 6 SMRT cells using the RS_HGAP Assembly.2 application with default settings and a genome size of 32 Mba [62]. The resulting assembly yielded 18 contigs, with five of these contigs containing characteristic telomeric (CCCTAA)_n_ or (TTAGGG)_n_ repeats on both ends and four of these contigs containing telomeric repeats on one end. The 5 contigs with telomeres on both ends were taken to be fully sequenced chromosomes. The remaining 13 contigs were evaluated for overlapping regions that could possibly be used to join the contigs. Two of these 13 contigs were discarded due to low coverage (<30×), three of these remaining contigs were found to have overlapping regions that allowed them to be joined into 1 supercontig and the remaining 8 contigs were found to also contain overlapping regions that would allow them to be joined into a second supercontig. These overlapping regions were evaluated by subjecting the entire genome of 5 chromosomes initially assembled by SMRT portal, plus the two manually curated supercontig chromosomes, to the SMRT portal resequencing protocol as a reference genome, along with manually evaluating these overlapping regions by evaluating the reads that spanned the overlapping regions. The resulting assembly was in 7 contigs, with each end of the contig terminating in a telomeric repeat sequence.

### Gene prediction, functional annotation and protein classification

Genome annotation was performed using the MAKER (version 2.31.8) pipeline using three *ab inito* gene prediction methods: Augustus trained for *Fusarium graminearum*, and GeneMark-ES and SNAP self-trained on the *C. militaris* genome [22, 23, 63–65]. Protein data from *Cordyceps brongniartii*, *C. militaris*, *Cordyceps confragosa*, *Ophiocordyceps sinensis*, *Ophiocordyceps unilateralis* and *Tolypocladium ophioglossoides* were used as protein evidence in MAKER. EST from *Cordyceps militaris* were downloaded from GeneBank and used as EST evidence in MAKER. Repeat elements were identified using Repeat Masker using the Repbase Library 20150807 [66]. A final set of consensus gene predictions was chosen using Exonerate [67]. The final gene set from MAKER was subjected to additional evidence modeling using Funannotate (0.7.0) [24]. The gene models were functionally annotated using the BLAST component of the Blast2GO software package and searching against the NCBI nr protein database (accessed July 2017) with the best hit being selected [68]. Gene families were established using the Interpro database using BlastProDOM, HMMPIR, HMMPfam, SuperFamily, SignalPHMM, HMMPanther [69]. The BLAST hits were mapped to the Gene Ontology database and KEGG analysis was carried out [70]. Secondary metabolite genes and gene clusters were predicted using both AntiSMASH, fungal version 4.0.0 and SMURF (accessed September 2016)[36, 38].

### Attempt at fruiting body production

Using the protocol outlined in Zheng et al. fruiting body production was attempted with *C. militaris* ATCC® 34164 [6].

### Attempt at militarinone production

Using the protocol in Schmidt et al. milititarone A–D production was attempted [44]. Specifically, precultures of *C. militaris* were used to inoculate 150 mL of medium (2% glucose, 2% neopeptone, 0.5% glycine, 0.2% K_2_HPO_4_, MgPO_4_-7H_2_O) in still 500 mL Erlenmeyer flasks at 25 °C. After 20 days the broth was removed and the mycelia were collected and freeze dried, then extracted with methanol. This methanol extract was treated with water (1.5 mL for 10 g of extract) and then partitioned in a 1:1:1:1 mixture of ethyl acetate/methanol/hexane/1% acetic acid. The lower phase was collected, concentrated, and then analyzed for militarinone production via UPLC-MS. No mass peaks corresponding to ionized militarinone A–D or sodium or acetate adducts of those natural products were apparent.

### Coverage and identity

Coverage and identity of *C. militaris* genes compared to genes in known biosynthetic clusters was determined using BLAST [61].

## Additional files

Additional file 1: Figure S1. Coverage across chromosomes. Coverage across chromosomes from SMRT analysis resequencing protocol assembly. (DOCX 1935 kb)

Additional file 2: Table S1. Predicted gene clusters in *C. militaris*. Predicted gene clusters are labeled, putative natural product class and the predicted length of each enzyme that is part of the putative cluster is given. (DOCX 106 kb)

## Abbreviations

HGAP: Hierarchical genome assembly process
MAT: Mating type
PacBio: Pacific Biosciences
PBcR: PacBio corrected reads

## References

[1] Lo H-C, Hsieh C, Lin F-Y, Hsu T-H (2013). A systematic review of the mysterious caterpillar fungus Ophiocordyceps sinensis in Dong-ChongXiaCao ( Dōng Chóng Xià Cǎo) and related bioactive ingredients. J Tradit Complement Med.

[2] Pontoppidan M-B, Himaman W, Hywel-Jones NL, Boomsma JJ, Hughes DP (2009). Graveyards on the move: the spatio-temporal distribution of dead Ophiocordyceps-infected ants. PLoS One.

[3] Paterson RRM (2008). Cordyceps – a traditional Chinese medicine and another fungal therapeutic biofactory?. Phytochemistry.

[4] Yue K, Ye M, Zhou Z, Sun W, Lin X (2013). The genus Cordyceps: a chemical and pharmacological review. J Pharm Pharmacol.

[5] Xia E-H, Yang D-R, Jiang J-J, Zhang Q-J, Liu Y, Liu Y-L (2017). The caterpillar fungus, Ophiocordyceps sinensis, genome provides insights into highland adaptation of fungal pathogenicity. Sci Rep.

[6] Zheng P, Xia Y, Xiao G, Xiong C, Hu X, Zhang S (2011). Genome sequence of the insect pathogenic fungus Cordyceps militaris, a valued traditional Chinese medicine. Genome Biol.

[7] Bushley KE, Raja R, Jaiswal P, Cumbie JS, Nonogaki M, Boyd AE (2013). The genome of tolypocladium inflatum: evolution, organization, and expression of the cyclosporin biosynthetic gene cluster. PLoS Genet.

[8] Agrawal Y, Khatri I, Subramanian S, Shenoy BD (2015). Genome sequence, comparative analysis, and evolutionary insights into Chitinases of Entomopathogenic fungus Hirsutella thompsonii. Genome Biol Evol.

[9] Adachi K, Chiba K (2007). FTY720 story. Its discovery and the following accelerated development of sphingosine 1-phosphate receptor agonists as immunomodulators based on reverse pharmacology. Perspect Med Chem.

[10] Traber R, Hofmann H, Kobel H (1989). Cyclosporins. New analogues by precursor directed biosynthesis. J Antibiot (Tokyo).

[11] Hoffmeister D, Keller NP (2007). Natural products of filamentous fungi: enzymes, genes, and their regulation. Nat Prod Rep.

[12] de Bekker C, Ohm RA, Loreto RG, Sebastian A, Albert I, Merrow M (2015). Gene expression during zombie ant biting behavior reflects the complexity underlying fungal parasitic behavioral manipulation. BMC Genomics.

[13] Keller NP, Turner G, Bennett JW (2005). Fungal secondary metabolism – from biochemistry to genomics. Nat Rev Microbiol.

[14] Rhoads A, Au KF (2015). PacBio sequencing and its applications. Genomics Proteomics Bioinformatics.

[15] Yandell M, Ence D (2012). A beginner’s guide to eukaryotic genome annotation. Nat Rev Genet.

[16] Berlin K, Koren S, Chin C-S, Drake JP, Landolin JM, Phillippy AM (2015). Assembling large genomes with single-molecule sequencing and locality-sensitive hashing. Nat Biotechnol.

[17] De Novo Assembly – Pacific Biosciences [Internet]. Available from: http://www.pacb.com/products-and-services/analytical-software/smrt-analysis/analysis-applications/de-novo-assembly/. Cited 8 Jun 2017.

[18] Simão FA, Waterhouse RM, Ioannidis P, Kriventseva EV, Zdobnov EM (2015). BUSCO: assessing genome assembly and annotation completeness with single-copy orthologs. Bioinformatics.

[19] Baker SE (2006). Aspergillus niger genomics: Past, present and into the future. Medical Mycology.

[20] Galagan JE, Calvo SE, Borkovich KA, Selker EU, Read ND, Jaffe D (2003). The genome sequence of the filamentous fungus Neurospora crassa. Nature.

[21] Stimberg N, Walz M, Schörgendorfer K, Kiick U (1992). Electrophoretic karyotyping from Tolypocladium inflatum and six related strains allows differentiation of morphologically similar species. Appl Microbiol Biotechnol.

[22] Campbell MS, Holt C, Moore B, Yandell M (2014). Genome annotation and curation using MAKER and MAKER-P. Curr Protoc Bioinformatics.

[23] Cantarel BL, Korf I, Robb SMC, Parra G, Ross E, Moore B (2008). MAKER: an easy-to-use annotation pipeline designed for emerging model organism genomes. Genome Res.

[24] Palmer J. Funannotate: pipeline for genome annotation [Internet]. Available from: https://github.com/nextgenusfs/funannotate/. Cited 1 May 2017.

[25] Cuomo CA, Guldener U, Xu J-R, Trail F, Turgeon BG, Di Pietro A (2007). The Fusarium graminearum genome reveals a link between localized polymorphism and pathogen specialization. Science.

[26] Quandt CA, Bushley KE, Spatafora JW (2015). The genome of the truffle-parasite Tolypocladium ophioglossoides and the evolution of antifungal peptaibiotics. BMC Genomics.

[27] Lennon MB, Suhadolnik RJ (1976). Biosynthesis of 3′-deoxyadenosine by Cordyceps militaris. Mechanism of reduction. Biochim Biophys Acta.

[28] Reichard P (2010). Ribonucleotide reductases: substrate specificity by allostery. Biochem Biophys Res Commun.

[29] Hu X, Zhang YJ, Xiao GH, Zheng P, Xia YL, Zhang XY (2013). Genome survey uncovers the secrets of sex and lifestyle in caterpillar fungus. Chin Sci Bull.

[30] Larriba E, Jaime MDLA, Carbonell-Caballero J, Conesa A, Dopazo J, Nislow C (2014). Sequencing and functional analysis of the genome of a nematode egg-parasitic fungus, Pochonia chlamydosporia. Fungal Genet Biol.

[31] Asai T, Yamamoto T, Oshima Y (2012). Aromatic Polyketide production in *Cordyceps indigotica*, an Entomopathogenic fungus, induced by exposure to a Histone Deacetylase inhibitor. Org Lett.

[32] Grudniewska A, Hayashi S, Shimizu M, Kato M, Suenaga M, Imagawa H (2014). Opaliferin, a new Polyketide from cultures of Entomopathogenic fungus *Cordyceps* sp. NBRC 106954. Org Lett.

[33] Khaokhajorn P, Samipak S, Nithithanasilp S, Tanticharoen M, Amnuaykanjanasin A (2015). Production and secretion of naphthoquinones is mediated by the MFS transporter MFS1 in the entomopathogenic fungus Ophiocordyceps sp. BCC1869. World J Microbiol Biotechnol.

[34] Varughese T, Rios N, Higginbotham S, Elizabeth Arnold A, Coley PD, Kursar TA (2012). Antifungal depsidone metabolites from Cordyceps dipterigena, an endophytic fungus antagonistic to the phytopathogen Gibberella fujikuroi. Tetrahedron Lett.

[35] Krasnoff SB, Reátegui RF, Wagenaar MM, Gloer JB, Gibson DM, Cicadapeptins I (2005). II: new Aib-containing peptides from the Entomopathogenic fungus *Cordyceps h eteropoda*. J Nat Prod.

[36] Weber T, Blin K, Duddela S, Krug D, Kim HU, Bruccoleri R (2015). antiSMASH 3.0--a comprehensive resource for the genome mining of biosynthetic gene clusters. Nucleic Acids Res.

[37] Fedorova ND, Moktali V, Medema MH (2012). Bioinformatics approaches and software for detection of secondary metabolic gene clusters. Methods Mol Biol.

[38] Khaldi N, Seifuddin FT, Turner G, Haft D, Nierman WC, Wolfe KH (2010). SMURF: genomic mapping of fungal secondary metabolite clusters. Fungal Genet Biol.

[39] Fisch KM (2013). Biosynthesis of natural products by microbial iterative hybrid PKS–NRPS. RSC Adv.

[40] Chooi Y-H, Tang Y (2012). Navigating the fungal polyketide chemical space: from genes to molecules. J Org Chem.

[41] Liu L, Zhang J, Chen C, Teng J, Wang C, Luo D (2015). Structure and biosynthesis of fumosorinone, a new protein tyrosine phosphatase 1B inhibitor firstly isolated from the entomogenous fungus Isaria fumosorosea. Fungal Genet Biol.

[42] Eley KL, Halo LM, Song Z, Powles H, Cox RJ, Bailey AM (2007). Biosynthesis of the 2-Pyridone tenellin in the insect pathogenic fungus Beauveria bassiana. Chembiochem.

[43] Heneghan MN, Yakasai AA, Williams K, Kadir KA, Wasil Z, Bakeer W (2011). The programming role of trans-acting enoyl reductases during the biosynthesis of highly reduced fungal polyketides. Chem Sci.

[44] Schmidt K, Gu W, Stoyanova S, Schubert B, Li Z, Hamburger M (2002). Militarinone A, a Neurotrophic Pyridone alkaloid from *Paecilomyces militaris*. Org Lett.

[45] Schmidt K, Riese U, Li Z, Hamburger M (2003). Novel Tetramic acids and Pyridone alkaloids, Militarinones B, C, and D, from the insect pathogenic fungus *Paecilomyces militaris*. J Nat Prod.

[46] D-C O, Kauffman CA, Jensen PR, Fenical W (2007). Induced production of Emericellamides a and B from the marine-derived fungus *Emericella* sp. in competing co-culture. J Nat Prod.

[47] Chiang Y-M, Szewczyk E, Nayak T, Davidson AD, Sanchez JF, Lo H-C (2008). Molecular genetic Mining of the Aspergillus Secondary Metabolome: discovery of the Emericellamide biosynthetic pathway. Chem Biol.

[48] Lukassen M, Saei W, Sondergaard T, Tamminen A, Kumar A, Kempken F (2015). Identification of the Scopularide biosynthetic gene cluster in Scopulariopsis brevicaulis. Mar Drugs.

[49] Niheik K, Itoh H, Hashimoto K, Miyairi K, Okuno T (1998). Antifungal Cyclodepsipeptides, W493 a and B, from *Fusarium* sp.: isolation and structural determination. Biosci Biotechnol Biochem.

[50] Sørensen JL, Sondergaard TE, Covarelli L, Fuertes PR, Hansen FT, Frandsen RJN (2014). Identification of the biosynthetic gene clusters for the Lipopeptides Fusaristatin a and W493 B in *Fusarium graminearum* and *F. pseudograminearum*. J Nat Prod.

[51] Vesonder RF, Tjarks LW, Rohwedder WK, Burmeister HR, Laugal JA (1979). Equisetin, an antibiotic from Fusarium equiseti NRRL 5537, identified as a derivative of N-methyl-2,4-pyrollidone. J Antibiot (Tokyo).

[52] Campbell CD, Vederas JC (2010). Biosynthesis of lovastatin and related metabolites formed by fungal iterative PKS enzymes. Biopolymers.

[53] Sims JW, Fillmore JP, Warner DD, Schmidt EW. Equisetin biosynthesis in Fusarium heterosporum. Chem Commun (Camb). 2005:186–8. 10.1039/b413523g 15724180

[54] Quast C, Pruesse E, Yilmaz P, Gerken J, Schweer T, Yarza P (2013). The SILVA ribosomal RNA gene database project: improved data processing and web-based tools. Nucleic Acids Res..

[55] Thompson JD, Gibson TJ, Higgins DG. Multiple sequence alignment using ClustalW and ClustalX. Curr Protoc Bioinforma. 2002. doi: 10.1002/0471250953.bi0203s00. 10.1002/0471250953.bi0203s00 18792934

[56] Saitou N, Nei M (1987). The neighbor-joining method: a new method for reconstructing phylogenetic trees. Mol Biol Evol.

[57] Tamura K, Nei M, Kumar S (2004). Prospects for inferring very large phylogenies by using the neighbor-joining method. Proc Natl Acad Sci U S A.

[58] Kumar S, Stecher G, Tamura K (2016). MEGA7: molecular evolutionary genetics analysis version 7.0 for bigger datasets. Mol Biol Evol.

[59] Toju H, Tanabe AS, Yamamoto S, Sato H (2012). High-coverage ITS primers for the DNA-based identification of ascomycetes and basidiomycetes in environmental samples. PLoS One.

[60] Schoch CL, Seifert KA, Huhndorf S, Robert V, Spouge JL, Levesque CA (2012). Nuclear ribosomal internal transcribed spacer (ITS) region as a universal DNA barcode marker for fungi. Proc Natl Acad Sci.

[61] Altschul SF, Gish W, Miller W, Myers EW, Lipman DJ (1990). Basic local alignment search tool. J Mol Biol.

[62] Chin C-S, Alexander DH, Marks P, Klammer AA, Drake J, Heiner C (2013). Nonhybrid, finished microbial genome assemblies from long-read SMRT sequencing data. Nat Methods.

[63] Stanke M, Keller O, Gunduz I, Hayes A, Waack S, Morgenstern B (2006). AUGUSTUS: ab initio prediction of alternative transcripts. Nucleic Acids Res.

[64] Borodovsky M, Lomsadze A. Eukaryotic gene prediction using GeneMark.hmm-E and GeneMark-ES. Curr Protoc Bioinforma. 2011. doi: 10.1002/0471250953.bi0406s35. 10.1002/0471250953.bi0406s35 PMC3204378 21901742

[65] Korf I (2004). Gene finding in novel genomes. BMC Bioinformatics.

[66] Smit, A. F, Hubley, R., Green P. RepeatMasker Home Page [Internet]. Repeat Masker 3.0. Available from: http://www.repeatmasker.org/. Cited 30 Sep 2015.

[67] Slater GSC, Birney E (2005). Automated generation of heuristics for biological sequence comparison. BMC Bioinformatics.

[68] O’Leary NA, Wright MW, Brister JR, Ciufo S, Haddad D, McVeigh R (2016). Reference sequence (RefSeq) database at NCBI: current status, taxonomic expansion, and functional annotation. Nucleic Acids Res.

[69] Hunter S, Apweiler R, Attwood TK, Bairoch A, Bateman A, Binns D (2009). InterPro: the integrative protein signature database. Nucleic Acids Res.

[70] Kanehisa M, Furumichi M, Tanabe M, Sato Y, Morishima K (2017). KEGG: new perspectives on genomes, pathways, diseases and drugs. Nucleic Acids Res.

