# Supplementary material for: Chromosome level assembly and secondary metabolite potential of the parasitic fungus Cordyceps militaris
Source: BMC Genomics. 2017 Nov 25;18:912. doi: 10.1186/s12864-017-4307-0 (PMC5702197; doi:10.1186/s12864-017-4307-0)
Supplement: Supplementary file 1 — Coverage across chromosomes. Coverage across chromosomes from SMRT analysis resequencing protocol assembly. (DOCX 1935 kb) [file 12864_2017_4307_MOESM1_ESM.docx]

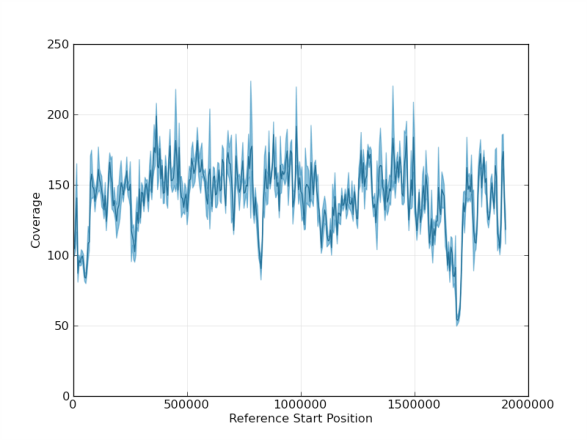

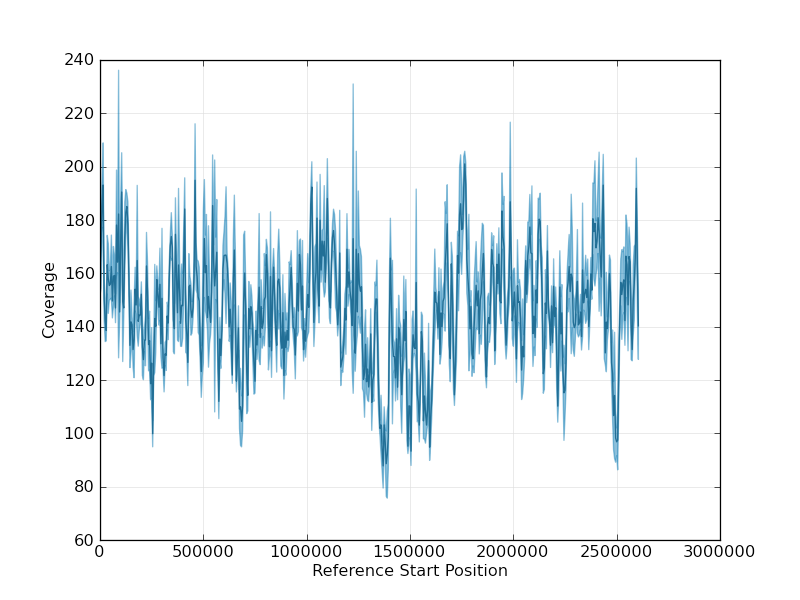

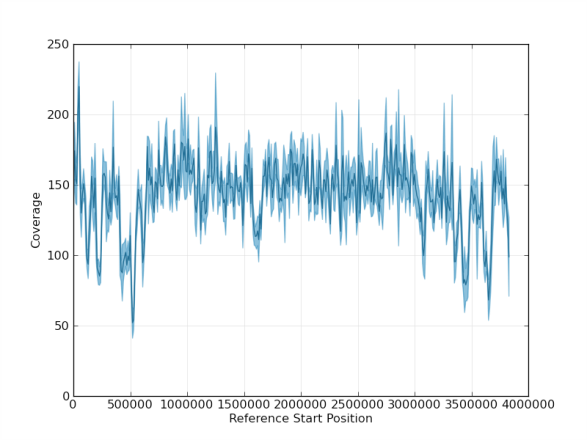

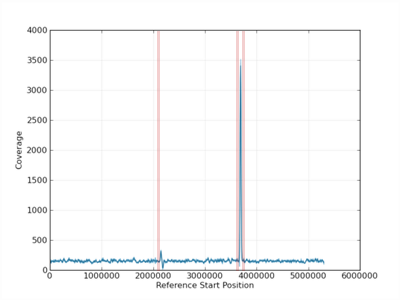

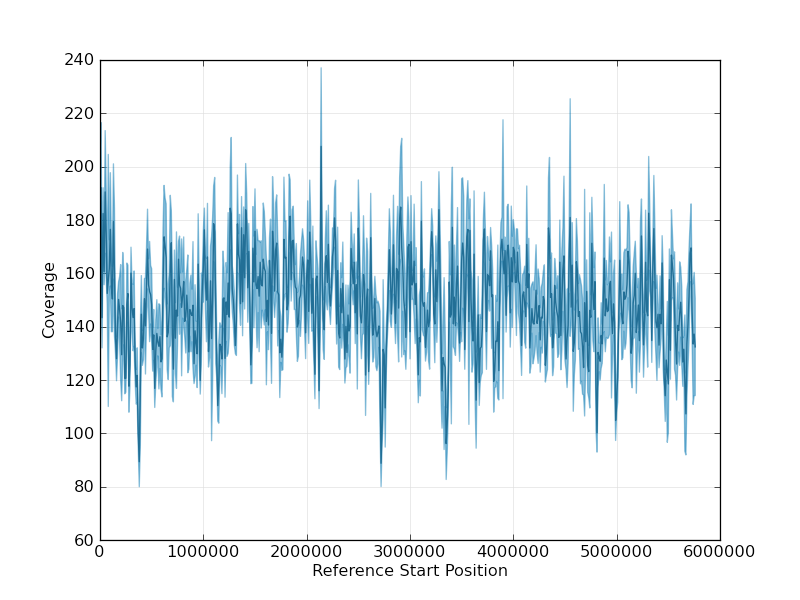

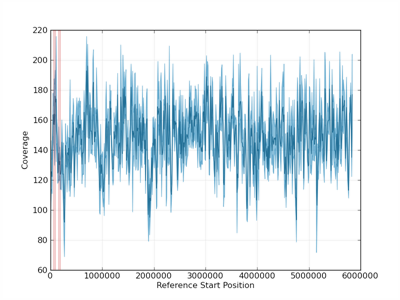

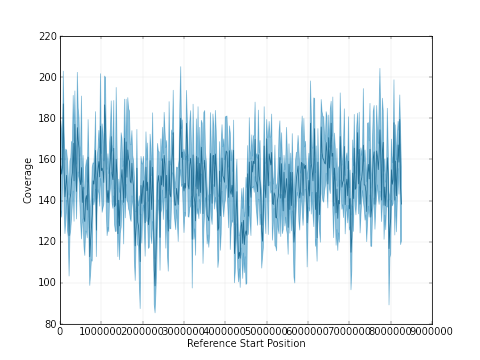


GD

F

E

D

C

B

A

Figure S1: Coverage across chromosomes from SMRT analysis resequencing protocol assembly. A. Chromosome I, B. Chromosome II, C. Chromosome III, D. Chromosome IV, E. Chromosome V, F. Chromosome VI, G. Chromosome VII. Areas where chromosomes were manually assembled, based on sequence homology are shown by the regions highlighted in pink (D, F). Chromosomes shown in A, B, C, E, and G were assembled in whole from telomere to telomere prior the manual curation and resequencing protocol.
