# Supplementary material for: Chromosome level assembly and secondary metabolite potential of the parasitic fungus Cordyceps militaris
Source: BMC Genomics. 2017 Nov 25;18:912. doi: 10.1186/s12864-017-4307-0 (PMC5702197; doi:10.1186/s12864-017-4307-0)
Supplement: Supplementary file 2 — Predicted gene clusters in C. militaris. Predicted gene clusters are labeled, putative natural product class and the predicted length of each enzyme that is part of the putative cluster is given. (DOCX 106 kb) [file 12864_2017_4307_MOESM2_ESM.docx]

**Predicted Gene Clusters in *C. militaris***

|  | **Gene Name** | **Annotation** | **Length (AA)** |
| --- | --- | --- | --- |
| I-1 T1PKS | A9K55_000411 | 60S ribosomal | 158 |
|  | A9K55_000412 | DNA glycosylase | 396 |
|  | A9K55_000413 | PAN domain containing | 127 |
|  | A9K55_000414 | C6 finger domain | 647 |
|  | A9K55_000415 | major facilitator superfamily | 536 |
|  | A9K55_000416 | AMP dependent | 597 |
|  | A9K55_000417 | hypothetical protein | 292 |
|  | A9K55_000418 | polyketide synthase | 2487 |
|  | A9K55_000419 | kinase-like domain | 282 |
|  | A9K55_000420 | kinase-like domain | 455 |
|  | A9K55_000421 | DNA repair | 295 |
|  |  |  |  |
| II-1 T1PKS | A9K55_000477 | major facilitator superfamily | 616 |
|  | A9K55_000478 | short chain dehydrogenase reductase | 324 |
|  | A9K55_000479 | carboxylesterase family | 583 |
|  | A9K55_000480 | conidial pigment biosynthesis oxidase | 724 |
|  | A9K55_000481 | hydroxyindole O-methyltransferase | 420 |
|  | A9K55_000482 | fungal transcriptional regulatory | 261 |
|  | A9K55_000483 | polyketide synthase | 2324 |
|  | A9K55_000484 | ATP-dependent bile acid permease | 1710 |
|  | A9K55_000485 | CFEM domain-containing | 349 |
|  | A9K55_000486 | P- phospholipid- flippase | 1542 |
|  | A9K55_000487 | homeoprotein | 626 |
|  |  |  |  |
| II-2 Other | A9K55_000491 | catalase peroxidase HPI | 795 |
|  | A9K55_000492 | major facilitator superfamily transporter | 618 |
|  | A9K55_000493 | serum paraoxonase arylesterase family | 436 |
|  | A9K55_000494 | hypothetical protein | 212 |
|  | A9K55_000495 | acyl- dehydrogenase | 431 |
|  | A9K55_000496 | epoxide hydrolase | 408 |
|  | A9K55_000497 | major facilitator superfamily transporter | 571 |
|  | A9K55_000498 | hypothetical protein | 473 |
|  | A9K55_000499 | alpha,alpha-trehalose glucohydrolase | 1007 |
|  | A9K55_000500 | carboxypeptidase cpdS | 523 |
|  | A9K55_000501 | hypothetical protein | 490 |
|  | A9K55_000502 | hypothetical protein | 122 |
|  | A9K55_000503 | hypothetical protein | 280 |
|  | A9K55_000504 | transcriptional regulator | 162 |
|  | A9K55_000505 | methyltransferase | 400 |
|  | A9K55_000506 | monooxygenase, putative | 395 |
|  | A9K55_000507 | para-nitrobenzyl esterase | 1336 |
|  |  |  |  |
| II-3 Terpene | A9K55_001091 | major facilitator superfamily transporter | 1265 |
|  | A9K55_001092 | hypothetical protein | 393 |
|  | A9K55_001093 | hypothetical protein | 82 |
|  | A9K55_001094 | kinase-like domain | 1447 |
|  | A9K55_001095 | floculation FLO1 | 358 |
|  | A9K55_001096 | integral peroxisomal membrane peroxin | 478 |
|  | A9K55_001097 | geranylgeranyl pyrophosphate synthetase | 462 |
|  | A9K55_001098 | hypothetical protein | 508 |
|  | A9K55_001099 | F-box cyclin | 67 |
|  | A9K55_001100 | short-chain dehydrogenase reductase family | 323 |
|  |  |  |  |
| II-4 T1PKS | A9K55_001109 | calpain-like protein | 873 |
|  | A9K55_001110 | COPII-coated vesicle | 376 |
|  | A9K55_001111 | hypothetical protein | 1188 |
|  | A9K55_001112 | kinase-like domain | 274 |
|  | A9K55_001113 | concanavalin A-like lectin glucanase | 238 |
|  | A9K55_001114 | kinase-like domain | 282 |
|  | A9K55_001115 | hypothetical protein | 373 |
|  | A9K55_001116 | kinase-like domain | 328 |
|  | A9K55_001117 | gamma-glutamyltranspeptidase periplasmic precursor | 1781 |
|  | A9K55_001118 | dipeptidyl peptidase III | 689 |
|  | A9K55_001119 | monocarboxylate permease | 475 |
|  | A9K55_001120 | dual oxidase 2 | 683 |
|  | A9K55_001121 | MFS transporter | 508 |
|  | A9K55_001122 | hypothetical protein | 757 |
|  | A9K55_001123 | kinase domain-containing | 349 |
|  | A9K55_001124 | hypothetical protein | 714 |
|  | A9K55_001125 | beta-ketoacyl synthase | 2141 |
|  | A9K55_001126 | NAD(P)-binding domain | 267 |
|  | A9K55_001127 | conidial pigment biosynthesis scytalone dehydratase Arp1 | 174 |
|  | A9K55_001128 | hypothetical protein | 94 |
|  | A9K55_001129 | hypothetical protein | 451 |
|  | A9K55_001130 | glycosyl transferase family | 321 |
|  | A9K55_001131 | ATG C terminal domain-containing | 2110 |
|  | A9K55_001132 | amine oxidase | 483 |
|  |  |  |  |
| III-1 T1PKS-NRPS | A9K55_001174 | C6 transcription | 738 |
|  | A9K55_001175 | major facilitator superfamily transporter | 514 |
|  | A9K55_001176 | dihydrodipicolinate synthetase | 308 |
|  | A9K55_001177 | acyltransferase | 227 |
|  | A9K55_001178 | major facilitator superfamily general substrate transporter | 441 |
|  | A9K55_001179 | C6 transcription | 545 |
|  | A9K55_001180 | dipeptidyl aminopeptidase acylaminoacyl-peptidase related | 439 |
|  | A9K55_001181 | hypothetical protein | 296 |
|  | A9K55_001182 | dihydroxyacetone kinase | 629 |
|  | A9K55_001183 | fungal transcription regulator | 610 |
|  | A9K55_001184 | ketose-bisphosphate class-II | 289 |
|  | A9K55_001185 | ribose 5-phosphate isomerase | 182 |
|  | A9K55_001186 | sorbose reductase | 285 |
|  | A9K55_001187 | vitamin H transporter | 469 |
|  | A9K55_001188 | MFS monocarboxylate | 449 |
|  | A9K55_001189 | C6 transcription | 775 |
|  | A9K55_001190 | polyketide synthase | 4151 |
|  | A9K55_001191 | ent-kaurene oxidase | 820 |
|  | A9K55_001192 | cytochrome P450 | 508 |
|  | A9K55_001193 | C6 zinc finger domain-containing | 72 |
|  | A9K55_001194 | C6 zinc finger domain-containing | 889 |
|  | A9K55_001195 | aspartic endopeptidase | 448 |
|  | A9K55_001196 | glycoside catalytic core | 228 |
|  | A9K55_001197 | integral membrane | 301 |
|  | A9K55_001198 | hypothetical protein | 554 |
|  | A9K55_001199 | phosphoglycerate kinase | 416 |
|  |  |  |  |
| III-2 Other | A9K55_001252 | fungal transcription regulator | 565 |
|  | A9K55_001253 | glucokinase | 427 |
|  | A9K55_001254 | urea active | 355 |
|  | A9K55_001255 | sodium solute symporter | 655 |
|  | A9K55_001256 | xenobiotic compound | 869 |
|  | A9K55_001257 | C6 zinc finger domain | 550 |
|  | A9K55_001258 | multicopper oxidase family | 638 |
|  | A9K55_001259 | hypothetical protein | 235 |
|  | A9K55_001260 | non-hemolytic phospholipase C | 641 |
|  | A9K55_001261 | hypothetical protein | 452 |
|  | A9K55_001262 | N-acetyltransferase | 187 |
|  | A9K55_001263 | 4-hydroxybenzoate polyprenyltransferase | 334 |
|  | A9K55_001264 | nucleoside phosphatase | 957 |
|  | A9K55_001265 | fatty acid hydroxylase | 356 |
|  | A9K55_001266 | hypothetical protein CCM_08328 | 191 |
|  | A9K55_001267 | BTB POZ fold domain containing | 293 |
|  | A9K55_001268 | siderophore iron transporter mirB | 573 |
|  | A9K55_001269 | transferase family | 1555 |
|  | A9K55_001270 | hypothetical protein | 361 |
|  | A9K55_001271 | kinesin family | 781 |
|  | A9K55_001272 | hypothetical protein | 501 |
|  | A9K55_001273 | amidase | 571 |
|  |  |  |  |
| III-3 Other | A9K55_001408 | histone-lysine N-methyltransferase | 584 |
|  | A9K55_001409 | BTB POZ fold domain containing | 255 |
|  | A9K55_001410 | hypothetical protein | 304 |
|  | A9K55_001411 | fructose-bisphosphate class II | 360 |
|  | A9K55_001412 | oxysterol binding | 415 |
|  | A9K55_001413 | caffeine-induced death | 192 |
|  | A9K55_001414 | acetyltransferase catalytic subunit | 819 |
|  | A9K55_001415 | sphingosine kinase | 497 |
|  | A9K55_001416 | extracellular membrane CFEM domain | 141 |
|  | A9K55_001417 | mitochondrial chaperone | 466 |
|  |  |  |  |
| III-4 NRPS | A9K55_001421 | hypothetical protein | 464 |
|  | A9K55_001422 | hypothetical protein | 300 |
|  | A9K55_001423 | 37S ribosomal | 215 |
|  | A9K55_001424 | mitochondrial inner membrane translocase subunit | 518 |
|  | A9K55_001425 | hypothetical protein | 160 |
|  | A9K55_001426 | sugar inositol transporter | 810 |
|  | A9K55_001427 | metallophosphoesterase | 446 |
|  | A9K55_001428 | hypothetical protein | 170 |
|  | A9K55_001429 | AMP-dependent synthetase ligase | 1397 |
|  | A9K55_001430 | carboxypeptidase A1 precursor | 411 |
|  | A9K55_001431 | dopa 4,5-dioxygenase | 173 |
|  | A9K55_001432 | 2OG-Fe(II) oxygenase family | 665 |
|  | A9K55_001433 | peptidase dimerization domain | 425 |
|  | A9K55_001434 | alcohol dehydrogenase zinc-containing | 364 |
|  | A9K55_001435 | cyanovirin-N | 122 |
|  | A9K55_001436 | conidiation-specific protein, putative | 262 |
|  | A9K55_001437 | alcohol acetyltransferase | 471 |
|  | A9K55_001438 | alginate lyase | 233 |
|  | A9K55_001439 | DUF221 domain | 1114 |
|  |  |  |  |
| III-5 NRPS | A9K55_001503 | ABC transporter | 147 |
|  | A9K55_001504 | ABC transporter | 963 |
|  | A9K55_001505 | membrane-associated progesterone receptor component | 194 |
|  | A9K55_001506 | acyl- N-acyltransferase | 207 |
|  | A9K55_001507 | glutamate carboxypeptidase | 901 |
|  | A9K55_001508 | inositol oxygenase | 346 |
|  | A9K55_001509 | chondroitin N-acetylgalactosaminyltransferase | 543 |
|  | A9K55_001510 | hypothetical protein | 386 |
|  | A9K55_001511 | amino acid polyamine transporter I | 538 |
|  | A9K55_001512 | multidrug resistance | 1285 |
|  | A9K55_001513 | 5 -nucleotidase | 628 |
|  | A9K55_001514 | non-ribosomal peptide synthetase | 1429 |
|  | A9K55_001515 | pyridoxal phosphate-dependent major region | 780 |
|  | A9K55_001516 | ankyrin repeat-containing domain | 338 |
|  | A9K55_001517 | hypothetical protein | 259 |
|  | A9K55_001518 | aminohydrolase | 327 |
|  | A9K55_001519 | hypothetical protein | 361 |
|  | A9K55_001520 | C6 zinc finger domain | 474 |
|  | A9K55_001522 | hypothetical protein | 388 |
|  | A9K55_001523 | kinase-like domain | 469 |
|  | A9K55_001524 | WW Rsp5 WWP domain | 211 |
|  | A9K55_001525 | cytochrome P450 | 563 |
|  | A9K55_001526 | hypothetical protein | 653 |
|  | A9K55_001527 | efflux pump antibiotic resistance | 582 |
|  | A9K55_001528 | cytochrome P450 | 555 |
|  | A9K55_001529 | cytochrome b5 | 193 |
|  | A9K55_001530 | zinc finger | 236 |
|  | A9K55_001531 | hypothetical protein | 148 |
|  | A9K55_001532 | hypothetical protein | 220 |
|  | A9K55_001533 | glyoxalase bleomycin resistance dioxygenase | 143 |
|  | A9K55_001534 | YCII-related protein | 117 |
|  | A9K55_001535 | hypothetical protein | 179 |
|  |  |  |  |
| IV-1 Terpene | A9K55_002256 | ankyrin repeat-containing | 592 |
|  | A9K55_002257 | hypothetical protein | 89 |
|  | A9K55_002258 | zinc finger | 863 |
|  | A9K55_002259 | ankyrin repeat-containing domain | 184 |
|  | A9K55_002260 | meiotic recombination spo11 | 301 |
|  | A9K55_002261 | lanosterol synthase | 740 |
|  | A9K55_002262 | ribulose-phosphate 3-epimerase | 261 |
|  | A9K55_002263 | SGT1 and CS domain containing | 513 |
|  | A9K55_002264 | taurine catabolism dioxygenase | 509 |
|  | A9K55_002265 | tRNA-splicing endonuclease subunit | 520 |
|  | A9K55_002266 | ceramide glucosyltransferase, putative | 530 |
|  |  |  |  |
| IV-2 Other | A9K55_002451 | hypothetical protein | 155 |
|  | A9K55_002452 | hypothetical protein | 687 |
|  | A9K55_002453 | hypothetical protein | 115 |
|  | A9K55_002454 | C6 transcription | 740 |
|  | A9K55_002455 | NRPS-like enzyme | 1231 |
|  | A9K55_002456 | MFS transporter | 526 |
|  | A9K55_002457 | small secreted | 139 |
|  | A9K55_002458 | CHY zinc finger domain | 685 |
|  | A9K55_002459 | kinase-like domain | 387 |
|  | A9K55_002460 | RTA1 domain | 292 |
|  | A9K55_002461 | C6 transcription factor | 453 |
|  | A9K55_002462 | T-complex 1 subunit epsilon | 562 |
|  | A9K55_002463 | elongation factor Tu GTP binding domain | 808 |
|  |  |  |  |
| IV-3 Other | A9K55_002584 | fungal specific transcription factor | 726 |
|  | A9K55_002585 | phosphoadenosine phosphosulfate reductase | 272 |
|  | A9K55_002586 | bax inhibitor family | 327 |
|  | A9K55_002587 | sphingosine N-acyltransferase lac1 | 472 |
|  | A9K55_002588 | copper amine oxidase 1 | 728 |
|  | A9K55_002589 | nicotinate-nucleotide pyrophosphorylase | 311 |
|  | A9K55_002590 | hypothetical protein | 363 |
|  | A9K55_002591 | hypothetical protein | 225 |
|  | A9K55_002592 | epoxide hydrolase | 593 |
|  | A9K55_002593 | TPR domain | 556 |
|  | A9K55_002594 | kinase domain | 420 |
|  |  |  |  |
| IV-4 NRPS | A9K55_002666 | hypothetical protein | 637 |
|  | A9K55_002667 | pathway-specific nitrogen regulator | 751 |
|  | A9K55_002668 | oxidoreductase-like protein | 254 |
|  | A9K55_002669 | xanthine phosphoribosyltransferase 1 | 204 |
|  | A9K55_002670 | hypothetical protein | 681 |
|  | A9K55_002671 | hypothetical protein | 330 |
|  | A9K55_002672 | cytochrome P450 alkane | 517 |
|  | A9K55_002673 | AMP-binding enzyme family | 1190 |
|  | A9K55_002674 | AMP-binding enzyme | 485 |
|  | A9K55_002675 | hypothetical protein | 626 |
|  | A9K55_002676 | intradiol ring-cleavage core | 351 |
|  | A9K55_002677 | ribosomal mitochondria | 396 |
|  | A9K55_002678 | MFS transporter | 157 |
|  | A9K55_002679 | phospho-2-dehydro-3-deoxyheptonate aldolase | 365 |
|  | A9K55_002680 | hypothetical protein | 286 |
|  |  |  |  |
| V-1 T1PKS-NRPS | A9K55_004080 | glucose-methanol-choline oxidoreductase | 563 |
|  | A9K55_004081 | glycoside family | 761 |
|  | A9K55_004082 | ATP-dependent Zn protease | 477 |
|  | A9K55_004083 | alpha beta hydrolase fold-1 | 387 |
|  | A9K55_004084 | hypothetical protein | 252 |
|  | A9K55_004085 | AMP-dependent synthetase ligase | 1420 |
|  | A9K55_004086 | hypothetical protein | 655 |
|  | A9K55_004087 | Alcohol dehydrogenase zinc-type | 360 |
|  | A9K55_004088 | Beta-ketoacyl synthase | 3881 |
|  | A9K55_004089 | LCCL domain-containing | 202 |
|  | A9K55_004090 | hypothetical protein | 501 |
|  | A9K55_004091 | cytochrome P450 | 527 |
|  | A9K55_004092 | glycosyl hydrolase family | 650 |
|  | A9K55_004093 | hypothetical protein | 206 |
|  | A9K55_004094 | molybdenum cofactor sulfurase | 108 |
|  | A9K55_004095 | acetamidase formamidase | 317 |
|  | A9K55_004096 | hypothetical protein | 87 |
|  | A9K55_004097 | GCN5-related N-acetyltransferase | 343 |
|  | A9K55_004098 | GATA transcription factor | 388 |
|  |  |  |  |
| V-2 Other | A9K55_004138 | C6 zinc finger domain | 481 |
|  | A9K55_004139 | hypothetical protein | 107 |
|  | A9K55_004140 | AAA family ATPase | 1548 |
|  | A9K55_004141 | short chain dehydrogenase | 313 |
|  | A9K55_004142 | dihydrofolate reductase | 436 |
|  | A9K55_004143 | ubiquinol-cytochrome C chaperone | 315 |
|  | A9K55_004144 | 3-oxoacyl-(acyl-carrier- ) synthase 2 | 428 |
|  | A9K55_004145 | small oligopeptide OPT family | 831 |
|  | A9K55_004146 | fungal transcriptional regulator | 748 |
|  | A9K55_004147 | hypothetical protein | 337 |
|  | A9K55_004148 | alcohol dehydrogenase | 348 |
|  | A9K55_004149 | flavin-containing amine | 463 |
|  | A9K55_004150 | C6 transcription factor | 743 |
|  | A9K55_004151 | product hydratase | 261 |
|  | A9K55_004152 | succinate-semialdehyde dehydrogenase | 496 |
|  | A9K55_004153 | general amino acid permease | 626 |
|  | A9K55_004154 | hypothetical protein | 352 |
|  |  |  |  |
| V-3 Indole | A9K55_004253 | U3 small nucleolar RNA associated | 445 |
|  | A9K55_004254 | 6-phosphofructo-2-kinase 1 | 669 |
|  | A9K55_004255 | cytidine deaminase | 423 |
|  | A9K55_004256 | Na(+) H(+) antiporter 2 | 565 |
|  | A9K55_004257 | zinc-binding oxidoreductase | 355 |
|  | A9K55_004258 | isoamyl alcohol | 670 |
|  | A9K55_004259 | dimethylallyl tryptophan synthase 1 | 940 |
|  | A9K55_004260 | fungal transcriptional regulator | 224 |
|  | A9K55_004261 | hypothetical protein | 284 |
|  | A9K55_004262 | hypothetical protein | 164 |
|  | A9K55_004263 | hypothetical protein | 240 |
|  | A9K55_004264 | actin cytoskeleton | 273 |
|  |  |  |  |
| V-4 NRPS | A9K55_004625 | PHD finger domain | 861 |
|  | A9K55_004626 | proteasome component Y13 | 251 |
|  | A9K55_004627 | PAP2 domain | 308 |
|  | A9K55_004628 | FF domain | 562 |
|  | A9K55_004629 | fungal specific transcription factor | 707 |
|  | A9K55_004630 | hypothetical protein | 86 |
|  | A9K55_004631 | L-ornithine N5-oxygenase | 666 |
|  | A9K55_004632 | nonribosomal siderophore peptide synthase | 4764 |
|  | A9K55_004633 | choline oxidase | 545 |
|  | A9K55_004634 | betaine aldehyde dehydrogenase | 642 |
|  | A9K55_004635 | hypothetical protein | 756 |
|  |  |  |  |
| V-5 T1PKS | A9K55_004807 | hypothetical protein | 349 |
|  | A9K55_004808 | serine threonine kinase | 638 |
|  | A9K55_004809 | hypothetical protein | 473 |
|  | A9K55_004810 | LMBR1-like conserved region | 711 |
|  | A9K55_004811 | hypothetical protein | 264 |
|  | A9K55_004812 | histone acetyltransferase type b catalytic | 477 |
|  | A9K55_004813 | pyrroline-5-carboxylate reductase | 315 |
|  | A9K55_004814 | asparagine synthase | 838 |
|  | A9K55_004815 | cytochrome P450 | 560 |
|  | A9K55_004816 | cytochrome b5 | 217 |
|  | A9K55_004817 | Beta-ketoacyl synthase | 2407 |
|  | A9K55_004818 | DUF341 domain | 284 |
|  | A9K55_004819 | epoxide hydrolase | 416 |
|  | A9K55_004820 | L-PSP endoribonuclease family | 150 |
|  | A9K55_004821 | hypothetical protein | 393 |
|  | A9K55_004822 | CTD kinase subunit gamma | 194 |
|  | A9K55_004823 | 50S ribosomal L12 | 189 |
|  | A9K55_004824 | midasin | 4911 |
|  | A9K55_004825 | isochorismatase domain-containing | 117 |
|  | A9K55_004826 | ubiquitin carboxyl-terminal hydrolase | 600 |
|  | A9K55_004827 | GDSL Lipase Acylhydrolase family | 264 |
|  | A9K55_004828 | SSU processome component | 1823 |
|  | A9K55_004829 | pyridine nucleotide-disulfide | 473 |
|  | A9K55_004830 | thiamine-phosphate pyrophosphorylase | 521 |
|  | A9K55_004831 | Zn(2)-C6 fungal-type DNA-binding domain | 609 |
|  |  |  |  |
| V-6 T1PKS-NRPS | A9K55_005035 | eukaryotic translation initiation factor 3 subunit | 232 |
|  | A9K55_005036 | DNA ligase | 865 |
|  | A9K55_005037 | hypothetical protein | 52 |
|  | A9K55_005038 | AP-1 complex subunit beta | 736 |
|  | A9K55_005041 | hypothetical protein | 308 |
|  | A9K55_005042 | hypothetical protein | 292 |
|  | A9K55_005039 | non-ribosomal peptide synthase | 4785 |
|  | A9K55_005040 | AMP dependent CoA ligase | 648 |
|  | A9K55_005043 | trichothecene 3-O-acetyltransferase | 485 |
|  | A9K55_005044 | beta-ketoacyl synthase | 2520 |
|  | A9K55_005045 | oligopeptide transporter | 779 |
|  | A9K55_005046 | mediated genome instability Rmi1 | 224 |
|  | A9K55_005047 | antiviral | 1421 |
|  | A9K55_005048 | Glycoside superfamily | 752 |
|  | A9K55_005049 | mitogen activated kinase kinase kinase | 1614 |
|  |  |  |  |
| V-7 T1PKS-NRPS | A9K55_005368 | amino acid polyamine transporter I | 522 |
|  | A9K55_005369 | ADF-like domain-containing | 146 |
|  | A9K55_005370 | dipeptidase domain containing | 516 |
|  | A9K55_005371 | Beta-ketoacyl synthase | 2465 |
|  | A9K55_005372 | Alcohol dehydrogenase zinc-type | 358 |
|  | A9K55_005373 | cytochrome P450 family | 526 |
|  | A9K55_005374 | LCCL domain-containing | 181 |
|  | A9K55_005375 | AMP-dependent synthetase ligase | 1514 |
|  | A9K55_005376 | hydroxylase | 623 |
|  | A9K55_005377 | hypothetical protein | 206 |
|  | A9K55_005378 | hypothetical protein | 355 |
|  | A9K55_005379 | hypothetical protein | 444 |
|  | A9K55_005380 | GDSL Lipase Acylhydrolase family | 706 |
|  | A9K55_005381 | hypothetical protein | 825 |
|  |  |  |  |
| VI-1 Other | A9K55_005583 | rho guanyl nucleotide exchange factor | 1728 |
|  | A9K55_005584 | UPF0041 domain | 188 |
|  | A9K55_005585 | UPF0041 domain | 148 |
|  | A9K55_005586 | NADH-ubiquinone oxidoreductase 105 kDa subunit | 103 |
|  | A9K55_005587 | hypothetical protein | 1118 |
|  | A9K55_005588 | thioredoxin-like fold | 137 |
|  | A9K55_005589 | ribosomal L14 | 147 |
|  | A9K55_005590 | nascent polypeptide-associated alpha subunit | 207 |
|  | A9K55_005591 | ATP synthase D chain | 173 |
|  | A9K55_005592 | ubiquitin-conjugating enzyme | 149 |
|  | A9K55_005593 | L-aminoadipate-semialdehyde dehydrogenase large | 1174 |
|  | A9K55_005594 | WD repeat | 1471 |
|  | A9K55_005595 | hypothetical protein | 163 |
|  | A9K55_005596 | c-14 sterol reductase | 496 |
|  | A9K55_005597 | hypothetical protein | 409 |
|  | A9K55_005598 | hypothetical protein | 129 |
|  | A9K55_005599 | RING-9 protein | 805 |
|  | A9K55_005600 | LUC7-like protein | 227 |
|  | A9K55_005601 | hypothetical protein | 286 |
|  | A9K55_005602 | hypothetical protein | 113 |
|  | A9K55_005603 | C6 transcription factor | 371 |
|  | A9K55_005604 | lanthionine synthetase | 388 |
|  | A9K55_005605 | hypothetical protein | 608 |
|  | A9K55_005606 | hypothetical protein | 320 |
|  | A9K55_005607 | DUF718 domain | 119 |
|  | A9K55_005608 | methylcrotonoyl- carboxylase beta chain | 572 |
|  | A9K55_005609 | acyl- dehydrogenase oxidase | 425 |
|  | A9K55_005610 | D-arabinitol dehydrogenase | 359 |
|  | A9K55_005611 | hypothetical protein | 421 |
|  |  |  |  |
| VI-2 T1PKS | A9K55_005631 | zinc finger dhhc domain containing | 887 |
|  | A9K55_005632 | peptidase | 105 |
|  | A9K55_005633 | woronin body major | 203 |
|  | A9K55_005634 | serologically defined colon cancer antigen | 1048 |
|  | A9K55_005635 | annexin | 771 |
|  | A9K55_005637 | methyltransferase | 519 |
|  | A9K55_005638 | tRNA Gly | 544 |
|  | A9K55_005639 | Beta-ketoacyl synthase | 2374 |
|  | A9K55_005640 | MFS multidrug | 585 |
|  | A9K55_005641 | stress responsive A B barrel domain | 117 |
|  | A9K55_005642 | hypothetical protein | 256 |
|  | A9K55_005643 | integral membrane | 213 |
|  | A9K55_005644 | ER to Golgi transport | 320 |
|  | A9K55_005645 | signal sequence receptor alpha subunit | 644 |
|  | A9K55_005646 | mid2-like cell wall stress sensor | 263 |
|  | A9K55_005647 | amidohydrolase domain | 337 |
|  | A9K55_005648 | hypothetical protein | 905 |
|  | A9K55_005649 | phospholipase C | 726 |
|  | A9K55_005650 | hypothetical protein | 355 |
|  | A9K55_005651 | dienelactone hydrolase family | 254 |
|  | A9K55_005652 | phosphopantetheinyl transferase | 292 |
|  | A9K55_005653 | intramembrane protease 2 | 579 |
|  | A9K55_005654 | transaldolase | 320 |
|  | A9K55_005655 | peptidyl prolyl cis-trans isomerase | 163 |
|  | A9K55_005656 | transcriptional activator | 373 |
|  | A9K55_005632 | acyl- N-acyltransferase | 352 |
|  | A9K55_005633 | glucokinase | 549 |
|  |  |  |  |
| VI-3 NRPS | A9K55_005710 | myo-inositol-1(or 4)-monophosphatase | 365 |
|  | A9K55_005711 | prolyl 4- alpha subunit | 242 |
|  | A9K55_005712 | RNA-directed RNA polymerase | 1877 |
|  | A9K55_005713 | MYB DNA-binding domain containing | 782 |
|  | A9K55_005714 | hypothetical protein | 872 |
|  | A9K55_005715 | Ras like GTPase | 542 |
|  | A9K55_005716 | ABC1 domain | 667 |
|  | A9K55_005717 | non-ribosomal peptide | 4455 |
|  | A9K55_005718 | benzoate 4-monooxygenase cytochrome P450 | 516 |
|  | A9K55_005719 | ABC transmembrane type | 1481 |
|  | A9K55_005720 | hypothetical protein | 515 |
|  | A9K55_005721 | CDP-alcohol phosphatidyltransferase | 420 |
|  | A9K55_005722 | yap1 redox domain | 325 |
|  | A9K55_005723 | TORC1 growth control complex subunit | 2010 |
|  | A9K55_005724 | hypothetical protein | 700 |
|  | A9K55_005725 | alpha-ketoglutarate-dependent sulfonate dioxygenase | 85 |
|  | A9K55_005726 | Glycoside superfamily | 269 |
|  | A9K55_005727 | hypothetical protein | 317 |
|  | A9K55_005728 | MFS multidrug transporter | 537 |
|  |  |  |  |
| VI-4 Terpene | A9K55_005902 | vacuolar aspartyl aminopeptidase | 590 |
|  | A9K55_005903 | cytochrome P450 | 70 |
|  | A9K55_005904 | geranylgeranyl pyrophosphate synthase | 682 |
|  | A9K55_005905 | hypothetical protein | 238 |
|  | A9K55_005906 | MFS multidrug | 551 |
|  | A9K55_005907 | cytochrome P450 oxidoreductase | 530 |
|  | A9K55_005908 | O-acetylhomoserine O-acetylserine sulfhydrylase | 512 |
|  | A9K55_005909 | cytochrome P450 | 526 |
|  | A9K55_005910 | transferase family | 541 |
|  | A9K55_005911 | benzoate 4-monooxygenase cytochrome P450 | 487 |
|  | A9K55_005912 | short chain dehydrogenase reductase family | 307 |
|  | A9K55_005913 | aristolochene synthase | 350 |
|  | A9K55_005914 | cytochrome P450 | 795 |
|  | A9K55_005915 | kinase-like domain | 404 |
|  | A9K55_005916 | secretory lipase, putative | 406 |
|  | A9K55_005917 | F-box cyclin | 412 |
|  | A9K55_005918 | formate dehydrogenase | 432 |
|  | A9K55_005919 | amino acid transporter | 620 |
|  |  |  |  |
| VI-5 Other | A9K55_006237 | major facilitator superfamily general substrate transporter | 569 |
|  | A9K55_006238 | endothiapepsin precursor | 429 |
|  | A9K55_006239 | tyrosinase precursor | 584 |
|  | A9K55_006240 | serine threonine- kinase | 563 |
|  | A9K55_006241 | fungal specific transcription factor | 745 |
|  | A9K55_006242 | glucose oxidase | 423 |
|  | A9K55_006243 | chromate ion transporter | 499 |
|  | A9K55_006244 | hypothetical protein | 594 |
|  | A9K55_006245 | AMP-dependent synthetase ligase | 1286 |
|  | A9K55_006246 | hypothetical protein | 69 |
|  | A9K55_006247 | hypothetical protein | 186 |
|  | A9K55_006248 | aspartyl protease | 359 |
|  | A9K55_006249 | hypothetical protein | 334 |
|  | A9K55_006250 | hypothetical protein | 151 |
|  | A9K55_006251 | tRNA pseudouridine synthase | 585 |
|  | A9K55_006252 | GABA permease | 536 |
|  | A9K55_006253 | phosphoribosylaminoimidazole-succinocarboxamide synthase | 326 |
|  | A9K55_006254 | peptidase alpha-lytic pro domain | 374 |
|  | A9K55_006255 | amino acid permease | 538 |
|  | A9K55_006256 | secreted protein | 203 |
|  | A9K55_006257 | NAD(P)-binding domain | 331 |
|  | A9K55_006258 | fungal transcriptional regulator | 400 |
|  |  |  |  |
| VI-6 NRPS | A9K55_006462 | hypothetical protein | 251 |
|  | A9K55_006463 | cytochrome c | 106 |
|  | A9K55_006464 | ribosome biogenesis ATPase | 732 |
|  | A9K55_006465 | spindle assembly checkpoint kinase | 267 |
|  | A9K55_006466 | polyketide synthesis O-methyltransferase | 303 |
|  | A9K55_006467 | ferric-chelate reductase | 743 |
|  | A9K55_006468 | SNF7 family | 205 |
|  | A9K55_006469 | hypothetical protein | 82 |
|  | A9K55_006470 | methyltransferase | 355 |
|  | A9K55_006471 | hypothetical protein | 147 |
|  | A9K55_006472 | hypothetical protein | 116 |
|  | A9K55_006473 | phosphatidylinositol 3- 4- catalytic | 812 |
|  | A9K55_006474 | FAD FMN-containing isoamyl alcohol oxidase - | 606 |
|  | A9K55_006475 | acyl- N-acyltransferase | 210 |
|  | A9K55_006476 | serine threonine- kinase | 589 |
|  | A9K55_006477 | hypothetical protein | 142 |
|  | A9K55_006478 | tetratricopeptide-like helical | 1098 |
|  | A9K55_006479 | hypothetical protein | 782 |
|  | A9K55_006480 | hypothetical protein | 205 |
|  | A9K55_006481 | rho GTPase activator Rga | 1121 |
|  | A9K55_006482 | non-ribosomal peptide synthetase | 1582 |
|  | A9K55_006483 | hypothetical protein | 113 |
|  | A9K55_006484 | monocarboxylate permease | 443 |
|  | A9K55_006485 | major facilitator superfamily general substrate transporter | 694 |
|  | A9K55_006486 | hypothetical protein | 335 |
|  | A9K55_006487 | hypothetical protein | 370 |
|  | A9K55_006488 | argininosuccinate lyase | 472 |
|  | A9K55_006489 | PBSP domain | 306 |
|  | A9K55_006490 | hypothetical protein | 187 |
|  | A9K55_006491 | HEAT repeat containing | 1089 |
|  | A9K55_006492 | NFX1-type zinc finger-containing | 1956 |
|  | A9K55_006493 | hypothetical protein | 472 |
|  | A9K55_006494 | hypothetical protein | 370 |
|  | A9K55_006495 | hypothetical protein | 681 |
|  | A9K55_006496 | zinc carboxypeptidase A 1 precursor | 758 |
|  | A9K55_006497 | MFS multidrug | 562 |
|  |  |  |  |
| VI-7 T1PKS | A9K55_006540 | hypothetical protein | 913 |
|  | A9K55_006541 | integral membrane | 437 |
|  | A9K55_006542 | guanyl-specific ribonuclease | 129 |
|  | A9K55_006543 | diacylglycerol o-acyltransferase | 519 |
|  | A9K55_006544 | DUF341 family | 253 |
|  | A9K55_006545 | Beta-ketoacyl synthase | 2386 |
|  | A9K55_006546 | amino acid | 577 |
|  | A9K55_006547 | hypothetical protein | 632 |
|  | A9K55_006548 | small-subunit Utp12 | 394 |
|  | A9K55_006549 | transcription regulator | 992 |
|  | A9K55_006550 | thiamin pyrophosphokinase-related | 335 |
|  | A9K55_006551 | peptidase subtilisin-related | 764 |
|  | A9K55_006552 | succinate dehydrogenase flavo subunit | 644 |
|  | A9K55_006553 | succinate dehydrogenase flavo subunit | 107 |
|  | A9K55_006554 | phospholipase carboxylesterase | 269 |
|  | A9K55_006555 | hypothetical protein | 520 |
|  | A9K55_006556 | hypothetical protein | 262 |
|  |  |  |  |
| VI-8 T1PKS | A9K55_006927 | DUF1711 domain | 265 |
|  | A9K55_006928 | short-chain dehydrogenase reductase SDR | 333 |
|  | A9K55_006929 | kinase-like domain | 321 |
|  | A9K55_006930 | ran-binding | 665 |
|  | A9K55_006931 | mitochondrial outer membrane | 298 |
|  | A9K55_006932 | SET domain | 362 |
|  | A9K55_006933 | hypothetical protein | 137 |
|  | A9K55_006934 | hypothetical protein | 630 |
|  | A9K55_006935 | kinase-like domain | 469 |
|  | A9K55_006936 | OTU domain-containing | 303 |
|  | A9K55_006937 | thiol methyltransferase | 291 |
|  | A9K55_006938 | short chain dehydrogenase reductase | 271 |
|  | A9K55_006939 | hypothetical protein | 123 |
|  | A9K55_006940 | serine threonine kinase | 281 |
|  | A9K55_006941 | beta-ketoacyl synthase | 2582 |
|  | A9K55_006942 | O-methyltransferase family 2 | 440 |
|  | A9K55_006943 | cytochrome P450 | 509 |
|  | A9K55_006944 | NAD(P)-binding domain | 287 |
|  | A9K55_006945 | alcohol dehydrogenase zinc-type | 354 |
|  | A9K55_006946 | flavin monooxygenase | 579 |
|  | A9K55_006947 | Xanthine uracil vitamin C permease | 570 |
|  | A9K55_006948 | 5-methylthioadenosine S-adenosylhomocysteine deaminase | 484 |
|  | A9K55_006949 | 2OG-Fe(II) oxygenase family | 319 |
|  | A9K55_006950 | hypothetical protein | 420 |
|  | A9K55_006951 | oxidoreductase domain containing | 455 |
|  | A9K55_006952 | kinase-like domain | 493 |
|  | A9K55_006953 | kinase domain | 228 |
|  | A9K55_006954 | hypothetical protein | 300 |
|  | A9K55_006955 | fungal transcriptional regulator | 478 |
|  | A9K55_006956 | major facilitator superfamily transporter | 687 |
|  | A9K55_006957 | hypothetical protein | 276 |
|  |  |  |  |
| VII-1 T1PKS-NRPS | A9K55_007002 | zinc finger domain-containing C2H2-type integrase | 1137 |
|  | A9K55_007003 | extracellular SCP domain | 219 |
|  | A9K55_007004 | hypothetical protein | 1395 |
|  | A9K55_007005 | late sexual development | 369 |
|  | A9K55_007006 | chitinase | 315 |
|  | A9K55_007007 | citrinin biosynthesis oxydoreductase | 235 |
|  | A9K55_007008 | beta-ketoacyl synthase | 3772 |
|  | A9K55_007009 | peptidase | 427 |
|  | A9K55_007010 | beta-ketoacyl synthase | 2403 |
|  | A9K55_007011 | N-terminal domain | 300 |
|  | A9K55_007012 | short-chain dehydrogenase reductase | 264 |
|  | A9K55_007013 | FAD binding type 2 | 580 |
|  | A9K55_007014 | cytochrome P450 | 559 |
|  | A9K55_007015 | MFS multidrug transporter | 519 |
|  |  |  |  |
| VII-2 Terpene | A9K55_007383 | hypothetical protein | 785 |
|  | A9K55_007384 | translin-associated factor | 254 |
|  | A9K55_007385 | mannosyltransferase (PIG-M) | 441 |
|  | A9K55_007386 | hypothetical protein | 601 |
|  | A9K55_007387 | oryzin precursor | 424 |
|  | A9K55_007388 | squalene synthetase | 466 |
|  | A9K55_007389 | DnaJ domain protein | 513 |
|  | A9K55_007390 | hypothetical protein | 763 |
|  | A9K55_007391 | ribosomal L19 | 994 |
|  | A9K55_007392 | long-chain fatty acid transporter | 639 |
|  | A9K55_007393 | cytochrome b5 | 347 |
|  | A9K55_007394 | DNA damage response | 782 |
|  | A9K55_007395 | hypothetical protein | 316 |
|  | A9K55_007396 | oxidoreductase domain containing | 265 |
|  |  |  |  |
| VII-3 NRPS | A9K55_008192 | zinc finger domain-containing PHD-finger | 740 |
|  | A9K55_008193 | hypothetical protein | 737 |
|  | A9K55_008194 | serine arginine repetitive matrix 1 | 543 |
|  | A9K55_008195 | histone deacetylase | 736 |
|  | A9K55_008196 | F-box domain | 346 |
|  | A9K55_008197 | brix domain-containing 2 | 313 |
|  | A9K55_008198 | chromo domain | 1213 |
|  | A9K55_008199 | peroxin | 360 |
|  | A9K55_008200 | PAPA-1-like conserved region | 407 |
|  | A9K55_008201 | hypothetical protein | 888 |
|  | A9K55_008202 | AMP-dependent synthetase ligase | 1808 |
|  | A9K55_008203 | AMP-dependent synthetase ligase | 780 |
|  | A9K55_008204 | siderophore biosynthesis | 452 |
|  | A9K55_008205 | hypothetical protein | 52 |
|  | A9K55_008206 | pyridine nucleotide-disulfide NAD-binding domain | 424 |
|  | A9K55_008207 | ABC transporter | 2356 |
|  | A9K55_008208 | indoleamine 2,3-dioxygenase | 481 |
|  | A9K55_008209 | DNA-directed RNA polymerase II polypeptide | 1211 |
|  | A9K55_008210 | cytochrome c oxidase assembly | 89 |
|  | A9K55_008211 | vacuolar ATP synthase subunit | 136 |
|  | A9K55_008212 | DUF1716 domain-containing | 578 |
|  |  |  |  |
| VII-4 NRPS | A9K55_008304 | cAMP-dependent kinase regulatory subunit | 1192 |
|  | A9K55_008305 | Gamma tubulin complex 3 | 869 |
|  | A9K55_008306 | acetyltransferase | 174 |
|  | A9K55_008307 | hypothetical protein | 92 |
|  | A9K55_008308 | hypothetical protein | 505 |
|  | A9K55_008309 | MFS transporter | 510 |
|  | A9K55_008310 | MFS transporter | 158 |
|  | A9K55_008311 | MFS transporter | 1111 |
|  | A9K55_008312 | hypothetical protein | 345 |
|  | A9K55_008313 | cytochrome P450 | 629 |
|  | A9K55_008314 | C-1-tetrahydrofolate synthase | 1078 |
|  | A9K55_008315 | peptide synthetase | 1266 |
|  | A9K55_008316 | 2OG-Fe(II) oxygenase family | 290 |
|  | A9K55_008317 | hypothetical protein | 341 |
|  | A9K55_008318 | eukaryotic translation initiation factor 3 subunit 2 | 337 |
|  | A9K55_008319 | chromatin remodelling complex ATPase chain ISW1 | 1115 |
|  | A9K55_008320 | hypothetical protein | 242 |
|  | A9K55_008321 | voltage-gated hydrogen channel 1 | 251 |
|  | A9K55_008322 | U3 snoRNP-associated Utp11 | 274 |
|  | A9K55_008323 | hypothetical protein | 632 |
|  | A9K55_008324 | hypothetical protein | 283 |
|  | A9K55_008325 | PAF acetylhydrolase | 555 |
|  |  |  |  |
| VII-5 T1PKS-NRPS | A9K55_008758 | mitochondrial carrier RIM2 | 388 |
|  | A9K55_008759 | fungal transcriptional regulator | 535 |
|  | A9K55_008760 | LSM domain-containing | 139 |
|  | A9K55_008761 | PAB-dependent poly(A)-specific ribonuclease subunit PAN3 | 639 |
|  | A9K55_008762 | acyl transferase acyl hydrolase lysophospholipase | 3955 |
|  | A9K55_008763 | MFS transporter | 555 |
|  | A9K55_008764 | fungal specific transcription factor domain | 685 |
|  | A9K55_008765 | hypothetical protein | 358 |
|  | A9K55_008766 | zinc-binding dehydrogenase family | 361 |
|  | A9K55_008767 | O-methyltransferase | 367 |
|  | A9K55_008768 | acyl- thioesterase | 734 |
|  | A9K55_008769 | acetyltransferase | 173 |
|  |  |  |  |
| VII-6 Other | A9K55_008935 | MFS transporter | 561 |
|  | A9K55_008936 | hypothetical protein | 158 |
|  | A9K55_008937 | basic leucine zipper | 442 |
|  | A9K55_008938 | antibiotic response | 183 |
|  | A9K55_008939 | hypothetical protein | 444 |
|  | A9K55_008940 | Glycoside superfamily | 529 |
|  | A9K55_008941 | NACHT and WD40 domain | 207 |
|  | A9K55_008942 | NADH-ubiquinone oxidoreductase | 188 |
|  | A9K55_008943 | hypothetical protein | 144 |
|  | A9K55_008944 | hypothetical protein | 413 |
|  | A9K55_008945 | Xanthine uracil vitamin C permease | 571 |
|  | A9K55_008946 | clavaminate synthase | 386 |
|  | A9K55_008947 | hypothetical protein | 173 |
|  | A9K55_008948 | hypothetical protein | 163 |
|  | A9K55_008949 | maltase | 600 |
|  | A9K55_008950 | sugar inositol transporter | 556 |
|  | A9K55_008951 | hypothetical protein | 3112 |
|  | A9K55_008952 | beta-galactosidase lacA | 1108 |
|  | A9K55_008953 | C6 transcription factor | 626 |
|  | A9K55_008954 | cation transport ATPase | 1102 |
|  | A9K55_008955 | Acyl- N-acyltransferase | 256 |
|  | A9K55_008956 | hypothetical protein | 433 |
|  | A9K55_008957 | ankyrin repeat | 619 |
|  | A9K55_008958 | AMP-dependent synthetase ligase | 633 |
|  | A9K55_008959 | hypothetical protein | 1129 |
|  | A9K55_008960 | haloacid dehalogenase-like | 296 |
|  | A9K55_008961 | hypothetical protein | 97 |
|  | A9K55_008962 | glycosyl family | 399 |
|  | A9K55_008963 | glycoside family | 541 |
|  | A9K55_008964 | amidohydrolase family | 546 |
|  | A9K55_008965 | hypothetical protein | 523 |
|  | A9K55_008966 | integral membrane | 487 |
|  | A9K55_008967 | hydantoinase carbamoylase | 863 |
|  | A9K55_008968 | metal ion resistance transporter | 1025 |
|  | A9K55_008969 | kinase subdomain-containing | 336 |
|  | A9K55_008970 | alcohol dehydrogenase | 675 |
|  | A9K55_008971 | LPS-induced tumor necrosis factor alpha factor | 224 |
|  | A9K55_008972 | oxalate decarboxylase | 424 |
|  | A9K55_008973 | selenium-binding protein | 485 |
|  | A9K55_008974 | acyl- thioesterase | 241 |
|  | A9K55_008975 | haloalkanoic acid | 232 |
|  | A9K55_008976 | gibberellin | 331 |
|  | A9K55_008977 | phosphoesterase family | 387 |
|  | A9K55_008978 | C6 transcription | 831 |
|  | A9K55_008979 | NAD-binding | 1046 |
|  | A9K55_008980 | hypothetical protein | 220 |
|  | A9K55_008981 | concanavalin A-like lectin glucanase | 853 |
|  | A9K55_008982 | mannosidase MsdS | 516 |
|  | A9K55_008983 | extracellular membrane | 195 |
|  | A9K55_008984 | acyltransferase | 519 |
|  | A9K55_008985 | von Willebrand domain containing | 947 |
|  | A9K55_008986 | nitrate reductase | 504 |
|  | A9K55_008987 | nitrate transporter | 509 |
|  | A9K55_008988 | amidophosphoribosyltransferase | 546 |
|  | A9K55_008989 | ABC transporter | 1595 |
|  |  |  |  |
| VII-7 Other | A9K55_009009 | MFS transporter | 588 |
|  | A9K55_009010 | hypothetical protein | 434 |
|  | A9K55_009011 | zinc RING FYVE PHD-type | 1617 |
|  | A9K55_009012 | cell division kinase | 377 |
|  | A9K55_009013 | Fes CIP4 domain-containing | 850 |
|  | A9K55_009014 | spindle assembly checkpoint component | 769 |
|  | A9K55_009015 | mannose-1-phosphate guanylyltransferase | 477 |
|  | A9K55_009016 | C-type cyclin | 640 |
|  | A9K55_009017 | phosphatidylinositol-3,4,5-trisphosphate 3-phosphatase | 521 |
|  | A9K55_009018 | non-ribosomal peptide synthetase | 1335 |
|  | A9K55_009019 | FAD binding | 578 |
|  | A9K55_009020 | asparaginyl-tRNA synthetase | 586 |
|  | A9K55_009021 | cytochrome P450 | 508 |
|  | A9K55_009022 | forkhead-associated | 542 |
|  | A9K55_009023 | ribosomal L1 | 378 |
|  | A9K55_009024 | palmitoyl- thioesterase precursor | 330 |
|  | A9K55_009025 | hypothetical protein | 77 |
|  | A9K55_009026 | transcription factor | 718 |
|  | A9K55_009027 | MFS transporter | 619 |
|  | A9K55_009028 | glycoside hydrolase beta alpha-barrel | 330 |
|  | A9K55_009029 | hypothetical protein | 184 |
|  | A9K55_009030 | UPF0145 domain-containing | 150 |
|  | A9K55_009032 | trehalase precursor | 682 |
|  | A9K55_009033 | cupin 2 domain-containing | 170 |
|  | A9K55_009034 | short chain dehydrogenase reductase family | 467 |
|  | A9K55_009035 | ankyrin repeat-containing domain | 1843 |
|  | A9K55_009036 | SNF2 family helicase ATPase | 888 |
|  | A9K55_009037 | rieske domain-containing | 240 |
|  | A9K55_009038 | tripeptidyl-peptidase 1 precursor | 564 |
|  | A9K55_009039 | amino acid polyamine transporter I | 567 |
|  | A9K55_009040 | kinase-like domain | 321 |

Table S1. Predicted gene clusters in *C. militaris*. Gene clusters are labeled, putative natural product class and the predicted length of each enzyme that is part of the putative cluster is given.
